# Bridging tradition and innovation: a constitution-guided framework for personalized blood pressure management in acute ischemic stroke

**DOI:** 10.3389/fmed.2025.1602274

**Published:** 2025-07-02

**Authors:** Xuran Zhang, Kegang Cao, Li Zhou, Jinxian Liu, Yufeng Ding

**Affiliations:** ^1^Dongzhimen Hospital, Beijing University of Chinese Medicine, Beijing, China; ^2^Beijing University of Chinese Medicine, Beijing, China

**Keywords:** acute ischemic stroke, blood pressure management, TCM constitution, personalized medicine, hypothesis and theory

## Abstract

**Background:**

Elevated blood pressure (BP) in acute ischemic stroke (AIS) significantly impacts clinical outcomes, yet optimal BP management remains contentious due to patient heterogeneity. Traditional Chinese medicine (TCM) constitution theory categorizes individuals into distinct physiological patterns, offering a novel framework to address this variability. This study integrates TCM constitutional theory into modern AIS care to propose a personalized BP management ‌hypothesis.

**Methods:**

A systematic review and meta-analysis were conducted across nine databases, including PubMed, Web of Science, Scopus, the Cochrane Library, ScienceDirect, the Chinese National Knowledge Infrastructure, Wanfang Data, VIP Database, and China Biology Medicine, covering publications up to January 2025. We analyzed TCM constitution distributions in AIS, hypertension, hypotension, AIS with hypertension, ischemic stroke (IS) with hypertension, and ischemic cerebrovascular disease with hypoperfusion, correlating constitutional types with clinical outcomes. We employed the Agency for Healthcare Research and Quality (AHRQ) checklist to evaluate the methodological quality of cross-sectional studies and utilized the Newcastle-Ottawa Scale (NOS) for quality assessment of cohort and case–control studies. Subgroup and sensitivity analyses were performed, and publication bias was assessed. A constitution-guided framework for BP management was developed through evidence synthesis.

**Results:**

Fifty-four studies were included in the study, with the majority being of moderate-to-high quality. The findings demonstrated that Phlegm-dampness, Qi-deficiency, Yin-deficiency, and Blood-stasis constitutions predominated in AIS patients with hypertension. Subgroup and sensitivity analyses confirmed the robustness of the results. Most analyses demonstrated no evidence of publication bias. Although several analyses indicated potential publication bias, the primary conclusions withstood the trim-and-fill adjustment and remained robust. A TCM constitution-based BP management hypothesis was proposed: patients with Phlegm-dampness or Blood-stasis constitutions may benefit from intensive BP control, whereas Qi-deficiency and Yin-deficiency types may require conservative strategies to mitigate hypoperfusion risks.

**Conclusion:**

This integration of TCM constitutional theory into AIS BP management provides a potential framework for advancing precision care to improve clinical outcomes in AIS patients. Further validation in multicenter cohorts and mechanistic exploration is warranted to enhance clinical applicability (Registration information: https://www.crd.york.ac.uk/PROSPERO2/view/CRD420250655689).

## 1 Introduction

Acute ischemic stroke (AIS) remains a leading cause of global morbidity and mortality (1, 2). Elevated blood pressure (BP) at onset, observed in approximately 75% of AIS patients (3), is strongly linked to adverse outcomes such as hemorrhagic transformation, cerebral edema, and long-term disability (4, 5). Current guidelines emphasize cautious BP management during the acute phase of AIS to balance cerebral perfusion and secondary injury risks (6–8). However, optimal BP targets remain contentious, as evidenced by conflicting results from landmark trials (e.g., ENCHANTED, OPTIMAL-BP) comparing intensive versus standard strategies (9–12). This ambiguity stems from marked patient heterogeneity in cerebral autoregulation, comorbid conditions, and therapeutic response, factors that remain inadequately addressed by conventional demographic or clinical stratification methods.

Traditional Chinese Medicine (TCM) constitution theory offers a novel framework to address this heterogeneity. Rooted in millennia of clinical observation, TCM constitution classification identifies distinct physiological and pathological patterns (nine TCM constitutions) that influence disease susceptibility and treatment efficacy (13). This study pioneers the integration of TCM constitution theory into modern BP management of AIS. By leveraging meta-analysis as an analytical tool, we systematically evaluate the distribution of TCM constitution types across AIS patients, hypertensive/hypotensive populations, and ischemic cerebrovascular cohorts with BP abnormalities. Through an extensive literature review and a step-by-step logical analysis, this investigation not only elucidates constitution-specific pathophysiological mechanisms underlying AIS with hypertension but also proposes a potential framework for personalized therapeutic strategies.

## 2 Methods

This meta-analysis was registered on the Prospective Register of Systematic Reviews (PROSPERO) under the code CRD420250655689 and conducted in accordance with Preferred Reporting Items for Systematic Reviews and Meta-Analyses (PRISMA) guidelines.

### 2.1 Deductive framework and analytical rationale

**Figure 1:**
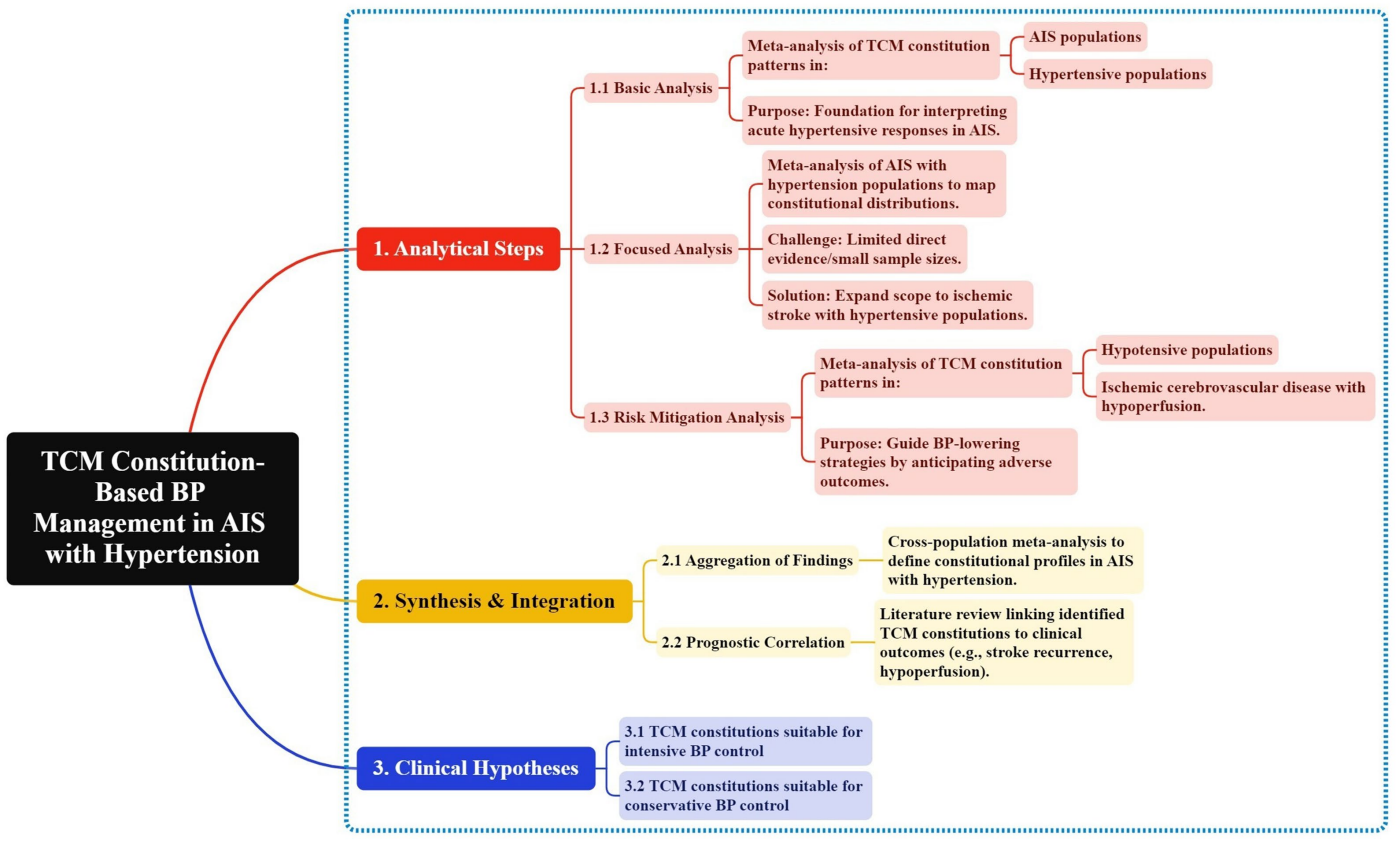
A TCM constitutions-guided framework for personalized BP management in AIS with hypertension.

To systematically elucidate the constitutional characteristics of AIS patients with hypertension and establish targeted BP management strategies, we developed a stepwise analytical framework grounded in evidence synthesis (Figure 1). First, we characterized TCM constitution distributions in foundational populations (AIS and hypertensive cohorts) to identify baseline traits linked to cerebrovascular pathology. Due to limited direct evidence in AIS with hypertensive population, we expanded analyses to broader ischemic stroke (IS) with hypertensive population. To address the risks of hypotension/hypoperfusion from aggressive BP lowering, we further examined TCM constitution patterns in hypotensive and ischemic cerebrovascular disease with hypoperfusion cohorts, identifying vulnerability traits. By synthesizing these findings and relevant research, we mapped the constitutional profiles in AIS with hypertensive patients and correlated them with clinical outcomes such as recurrence and hypoperfusion risks. This integrative approach enabled us to make targeted hypotheses: considering which TCM constitutions are, respectively, suitable for intensive BP control and conservative strategies.

### 2.2 Eligibility criteria

Studies were eligible for inclusion if they met the following criteria: (1) studies using the standardized TCM Constitution Classification and Determination criteria published by the China Association of Chinese Medicine (CACM) (14); (2) cohort studies, case–control studies, or cross-sectional studies; (3) studies providing sufficient data for calculating TCM constitution distribution in patients with AIS, hypertension, hypotension, AIS with hypertension, IS with hypertension, or ischemic cerebrovascular disease with hypoperfusion. Exclusion criteria were: (1) studies involving populations with other comorbidities; (2) studies analyzing only a single constitution type; (3) duplicate publications or secondary analyses of the same population; (4) reviews, commentaries, case reports, or animal studies; (5) studies with incomplete data reporting or inaccessible full texts.

### 2.3 Outcome measure

The outcome measure was the distribution of TCM constitution types in patients with AIS, hypertension, hypotension, AIS with hypertension, IS with hypertension, or ischemic cerebrovascular disease with hypoperfusion. To quantify this outcome, prevalence was used to describe the proportion of each constitution type within the study population, while odds ratios (ORs) were employed to compare the occurrence of specific constitution types between different patient groups. TCM constitution types were classified using the validated TCM Constitution Classification and Determination Criteria (ZYYXH/T157-2009) published by the CACM (14). (Supplementary material 1). This standardized tool defines nine constitution types: Balanced constitution (Type A) and eight unbalanced constitution types (Type B-I), including Qi-deficiency (Type B), Yang-deficiency (Type C), Yin-deficiency (Type D), Phlegm-dampness (Type E), Dampness-heat (Type F), Blood-stasis (Type G), Qi-stagnation (Type H), and Special Diathesis (Type I). Each participant completed the TCM Constitution Classification and Determination Questionnaire (Supplementary material 1), which consists of Likert-scale questions (5-point scoring) corresponding to the nine constitution domains. Raw scores were calculated by summing item responses for each constitution type. Conversion scores were derived using the formula:


$$\text{Conversion Score}=\frac{\text{Raw}\;\text{Score}-\text{Number of Items}}{\text{Number of Items}\times 4}\times 100$$

A Balanced constitution was defined as having a conversion score of ≥60, with all eight unbalanced constitution types simultaneously scoring <30, while unbalanced constitutions were categorized into three levels: “yes” for conversion scores ≥40, “tendency” for scores ranging from 30 to 39, and “no” for scores <30. The detailed questionnaire items and criteria for constitution classification are presented in Supplementary material 1.

### 2.4 Retrieval methods and screening process

A comprehensive search was conducted across nine electronic databases, including China National Knowledge Infrastructure (CNKI), Wanfang Data, VIP Database, China Biology Medicine (CBM), PubMed, Web of Science, Scopus, the Cochrane Library, and ScienceDirect. The search encompassed articles published in both Chinese and English languages up to January 2025. The included research types comprised cross-sectional study, case–control study, and cohort study. The search strategy employed a combination of Medical Subject Headings (MeSH) terms and free-text keywords, including but not limited to “acute ischemic stroke,” “acute cerebral infarction,” “constitution,” “ischemic stroke,” “cerebral infarction,” “hypertension,” “low blood pressure,” “hypoperfusion,” “traditional Chinese medicine,” “cohort study,” “case–control study,” and “cross-sectional study,” along with their respective Chinese equivalents. Complete search syntax exemplifying the PubMed strategy is detailed in Supplementary Tables 1–6 (Supplementary material 2), with database-specific adaptations implemented accordingly. The reference management process was implemented using EndNote 20 software. Two investigators independently performed a two-stage screening procedure involving sequential evaluation of titles, abstracts, and full-text documents. All stages of the literature search and selection process were subjected to reciprocal verification to determine eligible records. Any discrepancies were adjudicated through consensus discussions with senior reviewers.

### 2.5 Data extraction

The following information was systematically extracted from eligible studies: first author, publication year, study population, sample size, research design type, country of origin, and patient distribution across TCM constitution types. Two investigators independently performed data extraction using a pretested form and cross-checked the results for consistency, with discrepancies resolved through discussions.

### 2.6 Quality assessment

Quality assessment was conducted using standardized tools appropriate to each study design: cross-sectional studies were evaluated with the Agency for Healthcare Research and Quality (AHRQ) checklist (15), while cohort and case–control studies were appraised using the Newcastle-Ottawa Scale (NOS) (16). For the AHRQ checklist, each of the 11 items (e.g., clarity of data sources, inclusion/exclusion criteria, handling of confounding factors) was evaluated as “Yes,” “No,” “Unclear,” or “Not Applicable.” Scoring criteria were defined as follows: “Yes” responses were assigned 1 point, while “No,” “Unclear,” and “Not Applicable” received 0 point. For cross-sectional studies, Item 11 about follow-up was deemed “Not Applicable” due to the inherent design of such studies. Consequently, this item was excluded from the total score calculation, resulting in a maximum achievable score of 10. Study quality was classified based on established thresholds in the literature (17) studies fulfilling ≥7 scores were categorized as high quality, those scoring 4–6 as moderate quality, and those with ≤3 points as low quality.

The NOS evaluates studies across eight domains (e.g., cohort selection, comparability, outcome assessment), with a maximum score of 9. Quality classification followed established thresholds: studies scoring ≥7 points were classified as high quality, those with 5–6 points as moderate quality, and those with ≤4 points as low quality (18). Two researchers independently conducted quality assessments, followed by cross-verification of all entries. Any discrepancies were resolved through consensus discussions or, when necessary, adjudication by a third reviewer.

### 2.7 Statistical analysis

All statistical analyses were performed using Review Manager 5.3 and Stata software (version 14.0). Two types of analyses were conducted: (1) pooled prevalence estimates for single-group proportions, and (2) comparative analyses between groups using ORs. For synthesizing single-group prevalence estimates (e.g., TCM constitution distributions), pooled proportions were calculated using the logit transformation to stabilize variances of proportional data. Results were back-transformed to the original proportion scale via the inverse logistic transformation and reported as proportions (%) with 95% confidence intervals (CIs). Heterogeneity across studies was assessed using Cochran’s Q test (*p*-value) and the I^2^ statistic. A random-effects model was applied if significant heterogeneity was detected (*p* < 0.1 or I^2^ ≥ 50%); otherwise, a fixed-effects model was used. Comparative effect analyses (e.g., AIS with hypertension population vs. AIS without hypertension population) employed the inverse variance method for effect size calculation. Heterogeneity assessment and model selection criteria followed the same approach as described for prevalence analyses. All comparative results are expressed as ORs with 95% CIs.

Sensitivity analyses using leave-one-out cross-validation and subgroup analyses stratified by clinically relevant covariates were conducted to investigate potential sources of heterogeneity. When more than 10 studies were included in the analysis, funnel plots, Egger’s test, and Begg’s test would be employed to assess publication bias (19). If publication bias was detected, the trim-and-fill method would be applied to examine its impact (20).

## 3 Results

### 3.1 Literature selection and study characteristics

Fifty-four studies were ultimately included for analysis. The literature selection flowcharts are provided in Supplementary Figures 1–6 (Supplementary material 2). The characteristics of the studies are summarized in Table 1. The studies were conducted in China, with publication years spanning from 2009 to 2024, and sample sizes ranging from 9 to 26,579 participants. Research design types included cross-sectional (*n* = 40), cohort (*n* = 4), and case–control (*n* = 10). 28 studies have analyzed the distribution characteristics of TCM constitution in the AIS population. 13 studies focused on individuals with hypertension, three on those with hypotension, two on AIS patients with hypertension, 10 on IS with hypertension, and two on ischemic cerebrovascular disease with hypoperfusion. The distribution characteristics of the nine TCM constitutions for each population were also recorded in detail, respectively.

**Table 1:** The characteristics of the included studies.

Study ID	Research type	Study population	Sample size	Number of patients in each TCM constitution type								
				Balanced	Phlegm-dampness	Blood-stasis	Damp-heat	Qi-stagnation	Qi-deficiency	Yin-deficiency	Yang-deficiency	Special Diathesis
Li (21)	Cross-sectional	AIS	193	26	53	18	11	17	28	23	14	3
Cai (22)	Cohort	AIS	202	4	66	36	4	1	31	59	1	0
Liu (23)	Cross-sectional	AIS	120	16	17	4	8	6	33	17	18	1
Yang (24)	Cross-sectional	AIS	696	170	101	78	92	18	93	70	68	6
Yang (25)	Cross-sectional	AIS	120	2	31	9	1	4	41	25	7	0
Zhang et al. (26)	Cohort	AIS	168	14	32	5	8	8	57	34	10	0
Zhou et al. (27)	Cross-sectional	AIS	303	4	115	42	3	4	42	78	12	3
Yuan (28)	Cross-sectional	AIS	72	0	16	20	1	1	20	11	3	0
Huang (29)	Cross-sectional	AIS	180	11	55	38	7	5	36	13	15	0
Li (30)	Cross-sectional	AIS	248	26	183	146	40	108	94	49	33	18
Wang 2012 (31)	Cross-sectional	AIS	142	30	12	5	6	7	36	32	14	0
Li (32)	Cross-sectional	AIS	399	108	63	43	19	19	61	38	45	3
Cui (33)	Cross-sectional	AIS	153	42	21	7	12	15	18	11	19	8
Shi (34)	Cross-sectional	AIS	95	7	18	13	8	14	7	8	20	0
Liu (35)	Cohort	AIS	125	0	91	57	4	19	62	47	29	5
Zhen (36)	Case–control	AIS	237	NR	39	NR	NR	NR	69	46	NR	NR
Fu (37)	Cross-sectional	AIS	176	19	129	107	32	71	67	31	29	10
Qiu (38)	Cross-sectional	AIS	87	3	23	9	2	2	20	18	9	1
Yan (39)	Cross-sectional	AIS	289	16	56	55	21	21	43	40	29	8
Gao (40)	Cohort	AIS	302	18	67	43	19	31	58	35	22	9
Liang et al. (41)	Cross-sectional	AIS	116	17	17	3	8	6	30	17	16	2
Xu 2015 (42)	Cross-sectional	AIS	500	10	243	117	91	77	147	59	120	9
Li et al. (43)	Cross-sectional	AIS	178	5	20	61	4	10	62	19	29	1
Dong (44)	Cross-sectional	AIS	140	0	92	12	0	0	32	4	0	0
Zhang et al. (45)	Cross-sectional	AIS	124	15	19	4	8	6	34	19	18	1
Zou et al. (46)	Case–control	AIS	40	6	4	3	3	5	7	11	1	0
Li and Huang (47)	Cross-sectional	AIS	106	1	33	23	6	9	18	11	4	1
Li et al. (48)	Cross-sectional	AIS	200	12	46	36	37	16	15	29	9	0
Lin et al.(51)	Cross-sectional	Hypertension	322	80	87	18	14	8	45	30	34	6
Yang et al. (52)	Cross-sectional	Hypertension	1923	774	778	60	8	4	27	158	99	15
Li et al. (53)	Cross-sectional	Hypertension	2,592	449	138	889	78	36	109	471	377	45
Xiao et al.(54)	Cross-sectional	Hypertension	26,579	6,726	12,278	1,399	740	320	3,192	2,429	2,483	78
Lin et al. (55)	Case–control	Hypertension	300	47	34	4	41	9	74	15	70	6
Chen (56)	Cross-sectional	Hypertension	197	39	47	2	10	6	24	36	32	1
Zhang (57)	Case–control	Hypertension	174	65	71	4	2	6	8	6	10	2
Jin (58)	Case–control	Hypertension	250	36	68	48	NR	NR	NR	34	31	NR
Fan et al. (59)	Case–control	Hypertension	500	115	93	19	32	21	46	65	79	30
Zhang 2022 (60)	Cross-sectional	Hypertension	695	411	119	10	1	16	33	33	63	9
Wang and Hu 2023 (61)	Cross-sectional	Hypertension	515	134	217	3	17	12	21	55	54	2
Gao et al. (62)	Case–control	Hypertension	51	2	13	3	3	6	3	18	3	0
Li (63)	Case–control	Hypertension	1,041	35	305	49	130	11	50	272	189	NR
Zhou et al. (27)	Cross-sectional	AIS/IS with hypertension	227	3	89	30	1	4	31	58	8	3
Li (64)	Case–control	AIS/IS with hypertension	114	6	24	28	5	7	14	22	8	0
Yu et al. (65)	Cross-sectional	IS with hypertension	162	8	36	27	13	9	32	24	11	2
Wang (66)	Cross-sectional	IS with hypertension	139	6	47	18	8	9	28	10	11	2
Wang (67)	Case–control	IS with hypertension	47	2	16	11	1	0	13	2	2	0
Li (68)	Cross-sectional	IS with hypertension	527	0	182	54	5	9	160	98	19	0
Li (69)	Cross-sectional	IS with hypertension	224	3	43	65	30	0	76	5	1	1
Han (70)	Cross-sectional	IS with hypertension	215	15	45	7	23	17	46	58	4	NR
Song (71)	Cross-sectional	IS with hypertension	137	8	72	38	24	12	57	37	11	0
Wu (72)	Cross-sectional	IS with hypertension	273	13	53	37	22	28	63	29	17	11
Liu (73)	Cross-sectional	Hypotension	53	18	2	0	1	1	9	0	1	0
Wang and Miao (74)	Cross-sectional	Hypotension	210	8	57	29	19	26	162	46	128	6
Zhang (75)	Cross-sectional	Hypotension	108	0	13	0	1	0	7	83	4	0
Qiu (38)	Cross-sectional	Ischemic cerebrovascular disease with hypoperfusion	59	2	12	7	4	1	20	8	4	1
Chen (76)	Cross-sectional	Ischemic cerebrovascular disease with hypoperfusion	9	0	1	1	3	2	0	1	1	0

AIS, acute ischemic stroke; IS, ischemic stroke; TCM, traditional Chinese medicine; NR, not reported.

### 3.2 Quality evaluation of the included studies

**Figure 2:**
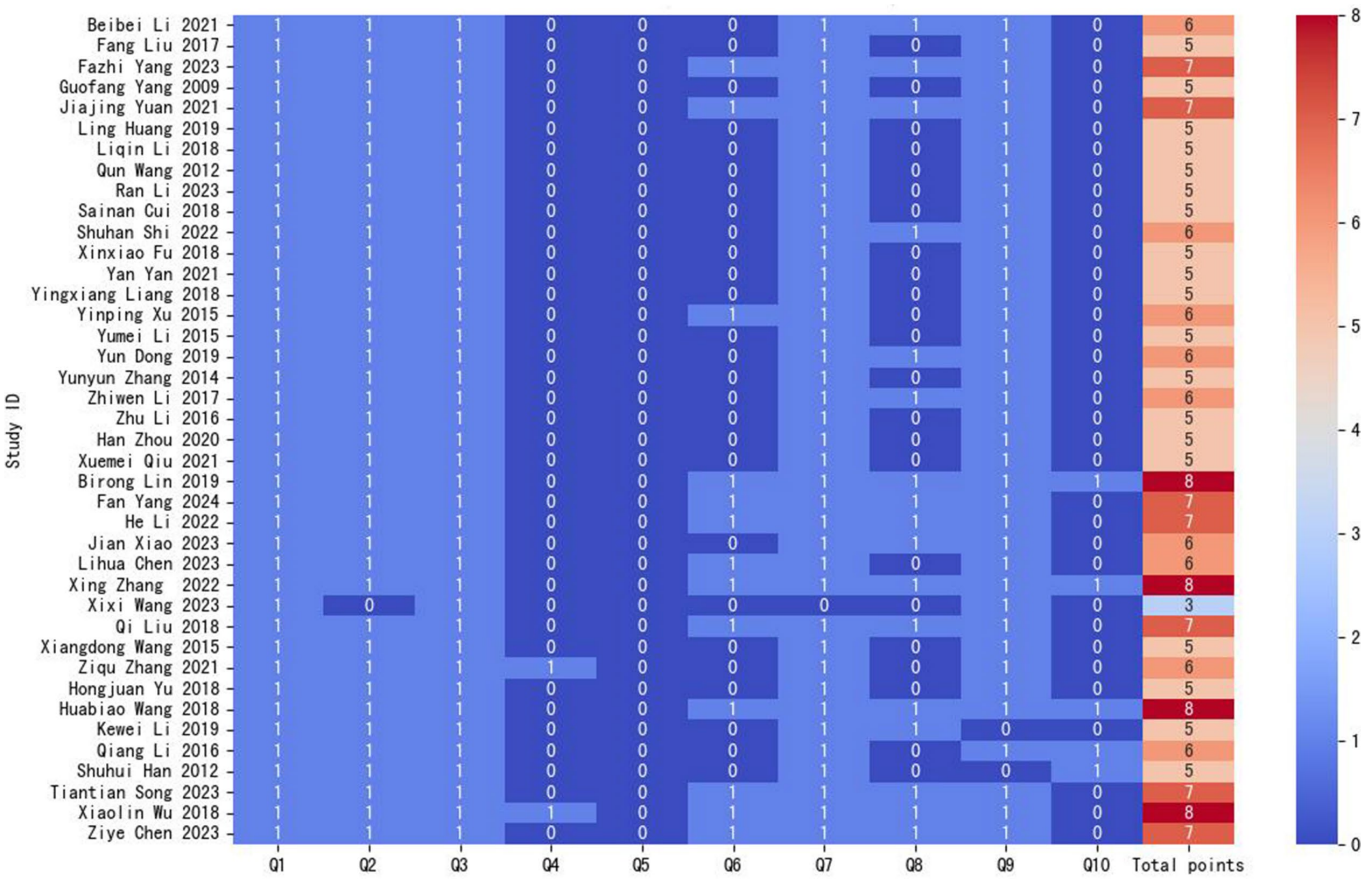
Results of the AHRQ scale assessment for cross-sectional studies. Q, question.

We assessed the quality of 40 cross-sectional studies using the AHRQ measurement tool (Figure 2). The results showed that 11 studies had a total score of 7 to 10 points and were considered to be of high quality. 28 studies had a total score of 4 to 6 points and were regarded as of moderate quality, and one study had a total score of 3 points and was considered to be of low quality. The majority of score deductions stemmed from insufficient reporting of consecutive enrollment status in non-population designs, unverified blinding procedures for subjective assessments, undocumented quality control measures for primary outcomes, or incomplete documentation of participant response rates and data completeness. The detailed scores for items of each study are presented in Supplementary Table 7 (Supplementary material 2).

**Figure 3:**
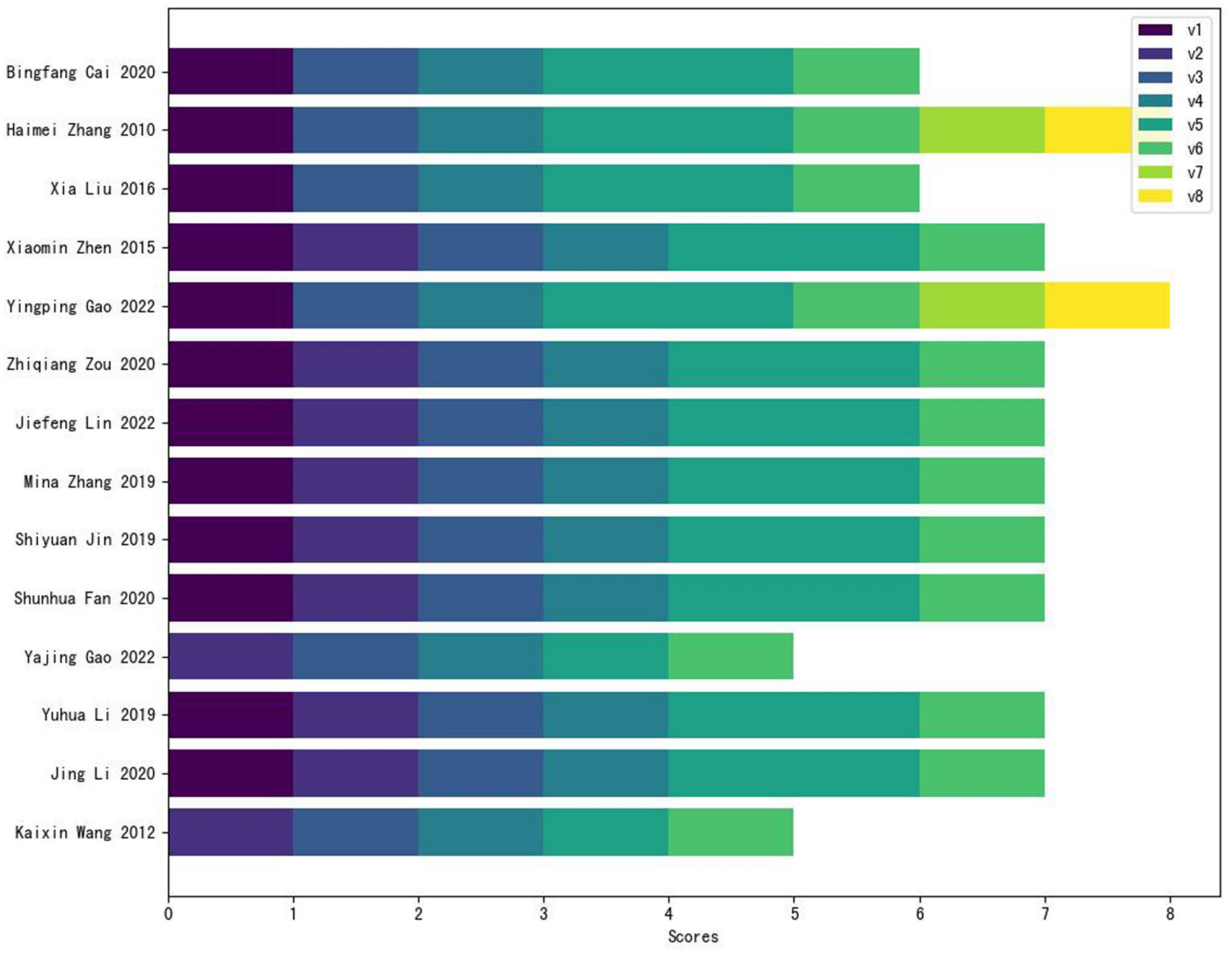
Results of the NOS scale assessment for cohort and case–control studies. V, variable.

The NOS scale was used to assess the quality of four cohort studies and 10 case–control studies (Figure 3). 10 studies scored 7–9 points (classified as high-quality), while 4 studies scored 5–6 points (categorized as moderate-quality). The primary reasons for score deductions across most studies included inadequate follow-up duration, failure to describe follow-up completeness, and insufficient documentation of non-exposed group recruitment sources. The detailed scores for 8 items of each study are presented in Supplementary Table 8 (Supplementary material 2).

### 3.3 Meta-analysis of TCM constitution patterns in study populations

Table 2 presents the meta-analysis results of TCM constitution distribution, whereas Table 3 compares TCM constitution types between the studied populations and other populations. The forest plots for the AIS population are presented in Figures 4, 5, with corresponding subgroup analysis forest plots provided in Figure 6. The forest plots for other meta-analyses and their subgroup analyses are presented in Supplementary Figures 7–19 (Supplementary material 2).

**Table 2:** Meta-analysis results of TCM constitution distribution in AIS, BP abnormalities, and ischemic cerebrovascular diseases with BP abnormalities populations.

Metrics		Balanced constitution	Phlegm-dampness constitution	Blood-stasis constitution	Damp-heat constitution	Qi-stagnation constitution	Qi-deficiency constitution	Yin-deficiency constitution	Yang-deficiency constitution	Special Diathesis constitution
AIS Population	Number of Studies	24	28	27	26	26	28	28	26	17
	Cumulative Cases	582	1,662	994	455	500	1,261	854	594	89
	Cumulative Sample Size	5,137	5,711	5,474	5,334	5,334	5,711	5,711	5,334	4,115
	Proportion	9%	36%	16%	7%	7%	28%	18%	11%	2%
	95% CI	[0.06, 0.13]	[0.25, 0.51]	[0.11, 0.24]	[0.05, 0.09]	[0.05, 0.12]	[0.23, 0.35]	[0.15, 0.22]	[0.09, 0.14]	[0.01, 0.03]
	Q Value	320.36	821.19	606.21	186.04	469.8	287.3	189.96	168.7	53.82
	I^2^/%	93	97	96	87	95	91	86	85	70
Hypertensive Population	Number of Studies	13	13	13	12	12	12	13	13	10
	Cumulative Cases	8,913	14,248	2,508	1,076	455	3,632	3,622	3,524	194
	Cumulative Sample Size	35,139	35,139	35,139	34,889	34,889	34,889	35,139	35,139	33,797
	Proportion	28%	33%	4%	4%	2%	7%	13%	13%	1%
	95% CI	[0.20, 0.39]	[0.22, 0.52]	[0.02, 0.09]	[0.02, 0.06]	[0.01, 0.03]	[0.05, 0.11]	[0.09, 0.18]	[0.10, 0.17]	[0.01, 0.03]
	Q Value	878.67	1532.45	2294.48	396.64	98.57	415.19	547.87	277.67	235.84
	I^2^/%	99	99	99	97	89	97	98	96	96
Hypotensive Population	Number of Studies	2	3	1	3	2	3	2	3	1
	Cumulative Cases	26	72	29	21	27	178	129	133	6
	Cumulative Sample Size	263	371	210	371	263	371	318	371	210
	Proportion	14%	15%	16%	3%	7%	37%	96%	11%	3%
	95% CI	[0.01, 1.78]	[0.05, 0.43]	[0.11, 0.24]	[0.01, 0.17]	[0.01, 0.44]	[0.03, 4.88]	[0.09, 10.82]	[0.01, 2.53]	[0.01, 0.07]
	Q Value	30.71	16.68	N/A	7.38	3.74	115.41	76.44	65.5	N/A
	I^2^/%	97	88	N/A	73	73	98	99	97	N/A
AIS with hypertension Population	Number of Studies	2	2	2	2	2	2	2	2	1
	Cumulative Cases	9	113	58	6	11	45	80	16	3
	Cumulative Sample Size	341	341	341	341	341	341	341	341	227
	Proportion	3%	42%	22%	2%	4%	15%	31%	5%	1%
	95% CI	[0.01, 0.12]	[0.18, 1.00]	[0.11, 0.47]	[0.00, 0.16]	[0.01, 0.13]	[0.11, 0.21]	[0.24, 0.40]	[0.03, 0.11]	[0.00, 0.04]
	Q Value	3.94	10.94	6.73	4.51	4.12	0.13	1.64	1.99	N/A
	I^2^/%	75	91	85	78	76	0	39	50	N/A
IS with hypertension Population	Number of Studies	9	10	10	10	8	10	10	10	5
	Cumulative Cases	64	607	315	132	95	520	343	92	19
	Cumulative Sample Size	1,538	2065	2065	2065	1794	2065	2065	2065	1,025
	Proportion	5%	40%	19%	6%	6%	31%	17%	5%	2%
	95% CI	[0.04, 0.06]	[0.29, 0.56]	[0.13, 0.28]	[0.04, 0.11]	[0.04, 0.09]	[0.23, 0.41]	[0.12, 0.25]	[0.04, 0.07]	[0.01, 0.04]
	Q Value	12.97	89.66	83.59	61.32	32.53	65.84	77.66	21.11	8.44
	I^2^/%	38	90	89	85	78	86	88	57	53
Ischemic cerebrovascular disease with hypoperfusion	Number of Studies	1	2	2	2	2	1	2	2	1
	Cumulative Cases	2	13	8	7	3	20	9	5	1
	Cumulative Sample Size	59	68	68	68	68	59	68	68	59
	Proportion	4%	24%	13%	18%	8%	51%	15%	8%	2%
	95% CI	[0.01, 0.14]	[0.13, 0.44]	[0.06, 0.28]	[0.03, 1.18]	[0.00, 1.17]	[0.30, 0.88]	[0.08, 0.31]	[0.03, 0.20]	[0.00, 0.12]
	Q Value	N/A	0.41	0	4.84	4.75	N/A	0.04	0.21	N/A
	I^2^/%	N/A	0	0	79	79	N/A	0	0	N/A

N/A, not applicable; AIS, acute ischemic stroke; IS, ischemic stroke.

**Table 3:** Comparison of TCM constitution types between study populations and other populations.

Metrics		Balanced constitution	Phlegm-dampness constitution	Blood-stasis constitution	Damp-heat constitution	Qi-stagnation constitution	Qi-deficiency constitution	Yin-deficiency constitution	Yang-deficiency constitution	Special Diathesis constitution
AIS Population VS General Population	Number of Studies	4	4	4	4	4	4	4	3	1
	Heterogeneity (I^2^/%)	80	46	0	68	56	79	55	63	N/A
	Model	Random	Fixed	Fixed	Random	Random	Random	Random	Random	Fixed
	Pooled OR	0.11	3.27	2.28	0.8	1	1.35	1.2	0.52	0.27
	95% CI	[0.05, 0.25]	[2.43, 4.40]	[1.69, 3.07]	[0.25, 2.60]	[0.33, 3.04]	[0.57, 3.19]	[0.68, 2.09]	[0.22, 1.24]	[0.02, 4.42]
	Z	5.09	7.79	5.41	0.37	0	0.67	0.63	1.48	0.92
	P	<0.00001	<0.00001	<0.00001	0.71	1	0.5	0.53	0.14	0.36
Hypertensive Population VS Non-hypertensive Population	Number of Studies	13	13	13	12	12	12	13	13	9
	Heterogeneity (I^2^/%)	97	86	53	12	28	64	84	83	46
	Model	Random	Random	Random	Fixed	Fixed	Random	Random	Random	Random
	Pooled OR	0.39	1.81	1.26	1.06	1.25	1.2	1.41	1.14	1.01
	95%CI	[0.28, 0.56]	[1.48, 2.21]	[1.03, 1.53]	[0.97, 1.16]	[1.09, 1.43]	[0.96, 1.51]	[1.09, 1.81]	[0.90, 1.44]	[0.69, 1.47]
	Z	5.26	5.75	2.28	1.36	3.15	1.59	2.66	1.07	0.05
	P	< 0.00001	< 0.00001	0.02	0.18	0.002	0.11	0.008	0.28	0.96
Hypotensive Population VS Normal BP Population	Number of Studies	1	2	N/A	1	1	2	1	2	N/A
	Heterogeneity (I^2^/%)	N/A	90	N/A	N/A	N/A	45	N/A	28	N/A
	Model	Fixed	Random	N/A	Fixed	Fixed	Fixed	Fixed	Fixed	N/A
	Pooled OR	0.88	1.29	N/A	0.4	0.46	3.06	3.24	1.26	N/A
	95%CI	[0.48, 1.64]	[0.02,69.40]	N/A	[0.05, 3.07]	[0.06, 3.65]	[1.41, 6.65]	[1.89, 5.55]	[0.37, 4.28]	N/A
	Z	0.39	0.13	N/A	0.89	0.73	2.83	4.29	0.37	N/A
	P	0.7	0.9	N/A	0.38	0.47	0.005	< 0.0001	0.71	N/A
AIS with hypertension Population VS AIS without hypertension Population	Number of Studies	2	2	2	2	1	2	2	2	N/A
	Heterogeneity (I^2^/%)	0	0	69	0	N/A	0	63	0	N/A
	Model	Fixed	Fixed	Random	Fixed	Fixed	Fixed	Random	Fixed	N/A
	Pooled OR	0.49	1.13	1.3	0.4	0.45	0.85	1.4	0.85	N/A
	95%CI	[0.19, 1.23]	[0.74, 1.71]	[0.52, 3.26]	[0.14, 1.10]	[0.17, 1.18]	[0.50, 1.45]	[0.62, 3.16]	[0.38, 1.90]	N/A
	Z	1.52	0.56	0.56	1.78	1.62	0.61	0.8	0.39	N/A
	P	0.13	0.57	0.57	0.08	0.11	0.54	0.42	0.7	N/A
IS with hypertension Population VS IS without hypertension Population	Number of Studies	8	8	8	7	5	8	8	8	3
	Heterogeneity (I^2^/%)	57	87	72	53	44	84	91	12	0
	Model	Random	Random	Random	Random	Fixed	Random	Random	Fixed	Fixed
	Pooled OR	0.5	2.15	1.1	1.18	1.26	1.05	0.66	0.66	0.78
	95%CI	[0.24, 1.03]	[1.02, 4.55]	[0.67, 1.81]	[0.62, 2.23]	[0.76, 2.08]	[0.57, 1.95]	[0.25, 1.73]	[0.45, 0.97]	[0.32, 1.89]
	Z	1.88	2.01	0.37	0.51	0.9	0.16	0.84	2.1	0.55
	P	0.06	0.04	0.71	0.61	0.37	0.88	0.4	0.04	0.58
Ischemic cerebrovascular disease with hypoperfusion VS Ischemic cerebrovascular disease with normoperfusion	Number of Studies	1	2	2	2	1	1	2	2	N/A
	Heterogeneity (I^2^/%)	N/A	0	68	0	N/A	N/A	0	30	N/A
	Model	Fixed	Fixed	Random	Fixed	Fixed	Fixed	Fixed	Fixed	N/A
	Pooled OR	1.93	0.7	0.95	1.69	0.98	1.95	0.48	1.01	N/A
	95%CI	[0.17, 21.90]	[0.30, 1.66]	[0.07, 12.30]	[0.35, 8.26]	[0.06, 16.09]	[0.63, 5.99]	[0.19, 1.17]	[0.24, 4.19]	N/A
	Z	0.53	0.81	0.04	0.65	0.01	1.16	1.62	0.01	N/A
	P	0.6	0.42	0.97	0.52	0.99	0.24	0.11	0.99	N/A

N/A, not applicable; AIS, acute ischemic stroke; IS, ischemic stroke.

**Figure 4:**
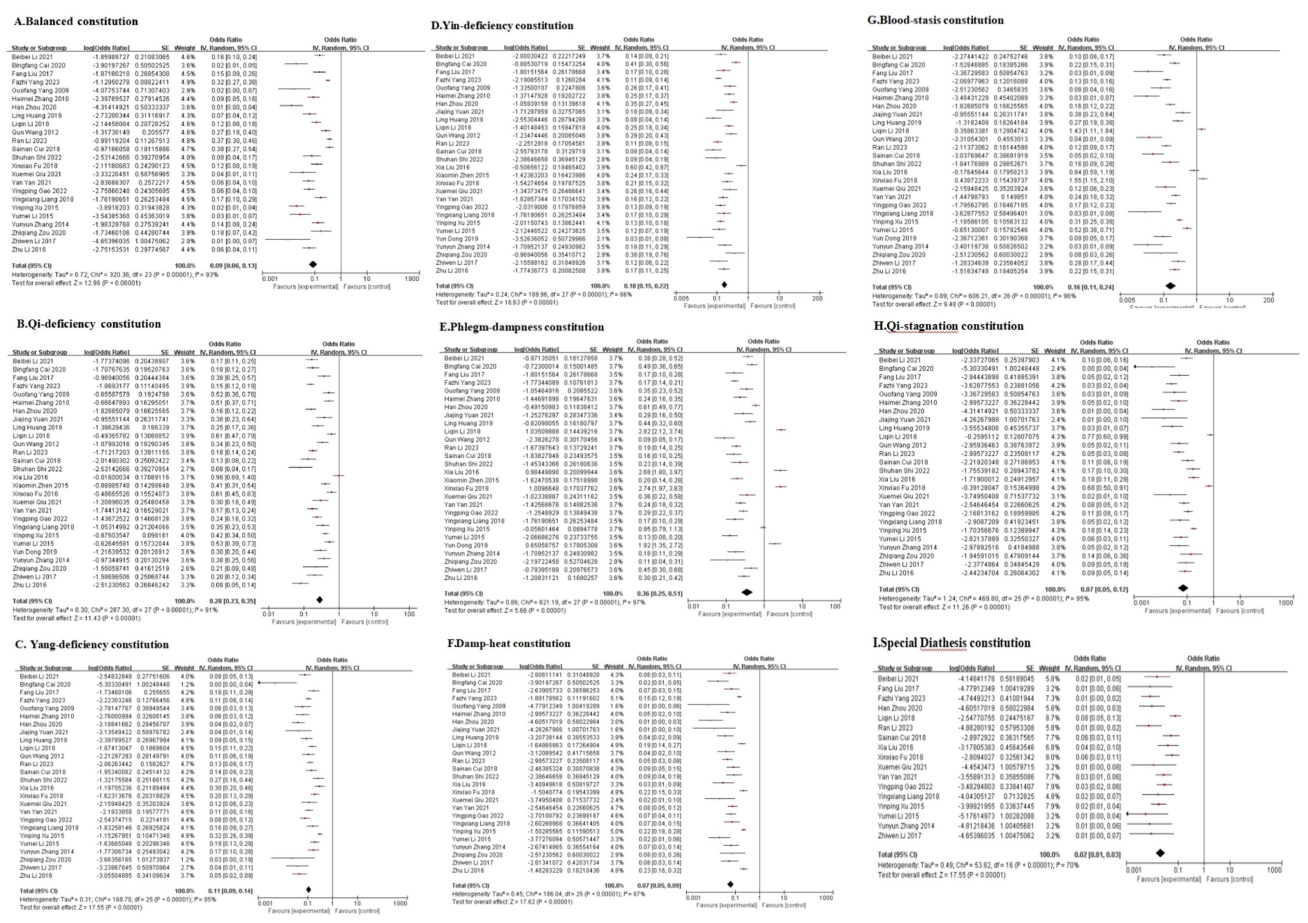
The forest plots of Balanced constitution **(A)**, Qi-deficiency constitution **(B)**, Yang-deficiency constitution **(C)**, Yin-deficiency constitution **(D)**, Phlegm-dampness constitution **(E)**, Damp-heat constitution **(F)**, Blood-stasis constitution **(G)**, Qi-stagnation constitution **(H)**, and Special Diathesis constitution **(I)** distributions in the AIS population.

**Figure 5:**
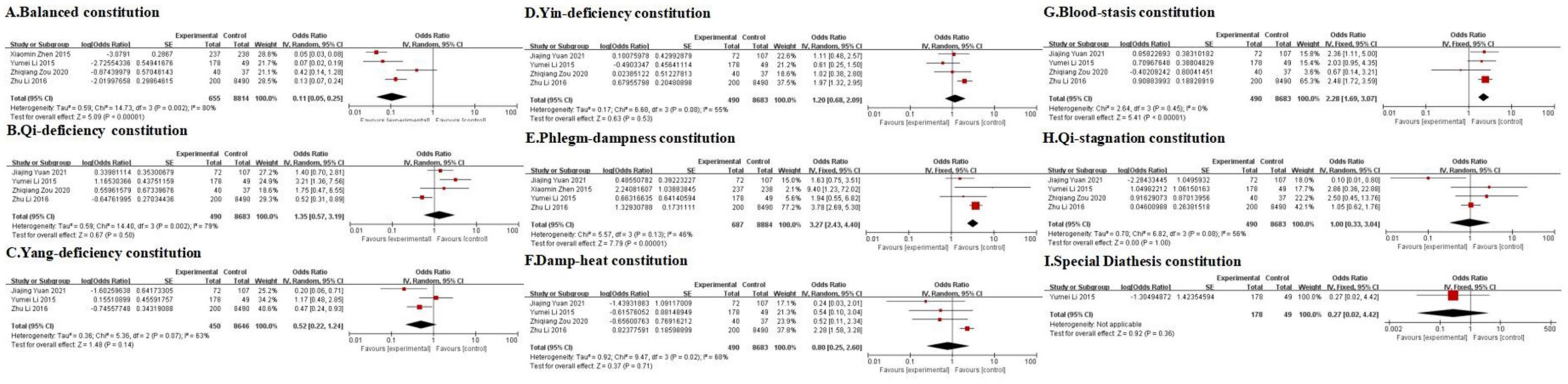
The forest plots comparing Balanced constitution **(A)**, Qi-deficiency constitution **(B)**, Yang-deficiency constitution **(C)**, Yin-deficiency constitution **(D)**, Phlegm-dampness constitution **(E)**, Damp-heat constitution **(F)**, Blood-stasis constitution **(G)**, Qi-stagnation constitution **(H)**, and Special Diathesis constitution **(I)** distributions between the AIS population and the general population.

**Figure 6:**
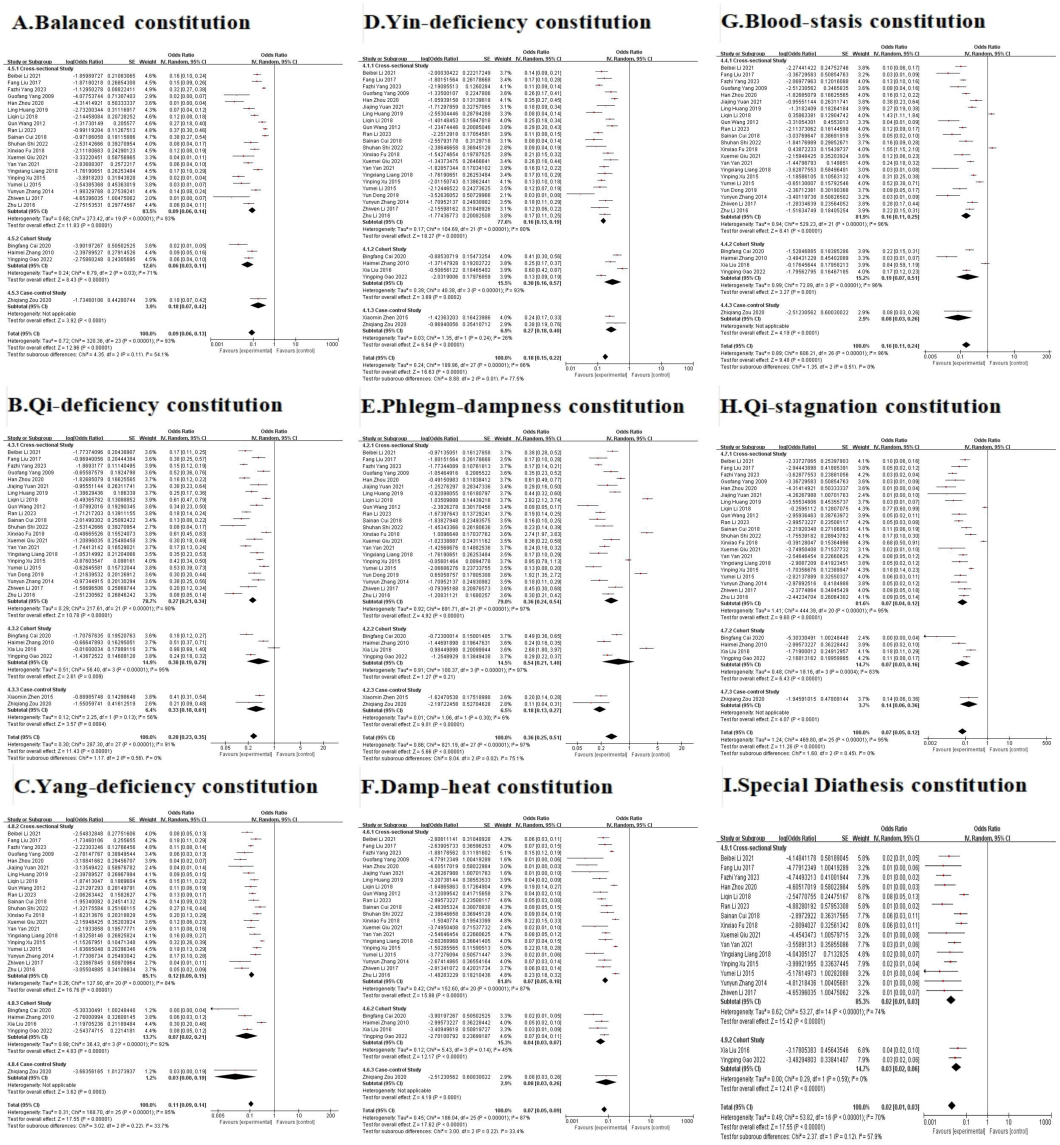
The subgroup analysis for Balanced constitution **(A)**, Qi-deficiency constitution **(B)**, Yang-deficiency constitution **(C)**, Yin-deficiency constitution **(D)**, Phlegm-dampness constitution **(E)**, Damp-heat constitution **(F)**, Blood-stasis constitution **(G)**, Qi-stagnation constitution **(H)**, and Special Diathesis constitution **(I)** distributions in the AIS population according to the study design.

#### 3.3.1 Meta-analysis of TCM constitution patterns in the AIS population

##### 3.3.1.1 Main findings

A total of 28 studies (21–48) were included in the analysis (Table 2, Figure 4). Due to significant heterogeneity among studies, a random-effects model was used for the meta-analysis. The results revealed that Phlegm-dampness (Figure 4E), Qi-deficiency (Figure 4B), Yin-deficiency (Figure 4D), and Blood-stasis constitutions (Figure 4G) predominated among AIS patients, each accounting for ≥15% of the study population. Five studies compared the distribution of TCM constitution types between AIS patients and the general population (Table 3, Figure 5), with a total sample size of 9,648 cases (727 AIS patients and 8,921 controls). The meta-analysis demonstrated that Phlegm-dampness constitution (OR = 3.27, 95% CI [2.43, 4.40], *p* < 0.00001) (Figure 5E) and Blood-stasis constitution (OR = 2.28, 95% CI [1.69, 3.07], p < 0.00001) (Figure 5G) were more prevalent in AIS patients. In contrast, the Balanced constitution (Figure 5A) was more common in the general population and served as a protective factor against AIS (OR = 0.11, 95% CI [0.05, 0.25], *p* < 0.00001). No statistically significant differences were observed in the distribution of other constitutional types between the two groups.

##### 3.3.1.2 Subgroup analysis

We conducted a subgroup analysis stratifying the 28 included studies by study design type (cross-sectional, cohort, and case–control). (Figure 6). Following subgroup analysis, heterogeneity decreased for seven of the nine TCM constitutions examined (Figures 6A,B,D–F,H,I). This suggests that the study design contributed to the observed high heterogeneity. While the proportion of specific constitutions within the AIS population varied across study types, the Phlegm-dampness (Figure 6E), Qi-deficiency (Figure 6B), Yin-deficiency (Figure 6D), and Blood-stasis constitutions (Figure 6G) consistently each accounted for ≥15% of cases across most studies. Conversely, the other five constitutions each represented <15% in most design categories. This confirms that Phlegm-dampness, Blood-stasis, Qi-deficiency, and Yin-deficiency constitutions are the predominant constitutions in AIS patients, aligning with the pooled meta-analysis results.

Considering the potential influence of disease severity and stroke subtype on TCM constitution distribution, we analyzed these factors. Only one study (21) reported on disease severity, specifically including mild-to-moderate AIS patients with a National Institutes of Health Stroke Scale (NIHSS) score <15. The prevalent constitutions identified—Phlegm-dampness (27.46%), Qi-deficiency (14.51%), Yin-deficiency (11.92%), and Blood-stasis (9.33%)—were consistent with the pooled meta-analysis findings. Similarly, only one study (44) assessed stroke subtypes, focusing exclusively on the large artery atherosclerosis (LAA) subtype. The top four constitutions were Phlegm-dampness (65.71%), Qi-deficiency (22.86%), Blood-stasis (8.57%), and Yin-deficiency (2.86%), again mirroring the primary meta-analysis results.

Collectively, these subgroup analyses reinforce the robustness of the conclusion that Phlegm-dampness, Blood-stasis, Qi-deficiency, and Yin-deficiency are the most prevalent constitutions in AIS across diverse study designs. It further suggests this pattern holds within the limited available data for mild-to-moderate disease severity and the LAA stroke subtype.

#### 3.3.2 Meta-analysis of TCM constitution patterns in the hypertensive population

##### 3.3.2.1 Main findings

Two studies have explored the distribution patterns of TCM constitutions among hypertensive populations (49, 50). A meta-analysis of 10 studies published before May 31, 2018, was conducted (49), revealing significant associations between hypertension and specific constitutional types. The analysis demonstrated a higher prevalence of Phlegm-dampness, Yin-deficiency, Qi-deficiency, and Blood-stasis constitutions in the hypertensive population, while a Balanced constitution showed protective effects (*p* < 0.05). These findings were further corroborated by another study (50), which included 13 case–control studies published before January 2019. The study identified constitutional profiles where Phlegm-dampness, balanced, Yin-deficiency, and Qi-deficiency types each accounted for >15% of cases. Compared with the general population, Phlegm-dampness, Qi-deficiency, and Yin-deficiency were found to be risk factors for hypertension, while the Balanced constitution was a protective factor, with all differences being statistically significant (*p* < 0.05).

To update and extend these findings, we performed a supplementary meta-analysis incorporating studies published between January 2019, and January 2025. Thirteen studies (51–63) revealed that Phlegm-dampness, balanced, Yin-deficiency, Qi-deficiency, and Yang-deficiency constitutions were prevalent in hypertensive individuals (Table 2, Supplementary Figure 7). These studies also compared the TCM constitution distribution between hypertensive (*n* = 35,139) and non-hypertensive (*n* = 40,003) populations. The meta-analysis showed that hypertensive individuals were more likely to exhibit Phlegm-dampness (OR = 1.81, 95% CI [1.48, 2.21], *p* < 0.00001), Yin-deficiency (OR = 1.41, 95% CI [1.09, 1.81], *p* = 0.008), Blood-stasis (OR = 1.26, 95% CI [1.03, 1.53], *p* = 0.02), and Qi-stagnation constitutions (OR = 1.25, 95% CI [1.09, 1.43], *p* = 0.002). In contrast, the Balanced constitution (OR = 0.39, 95% CI [0.28, 0.56], *p* < 0.00001) was more common in non-hypertensive individuals. No statistically significant differences were observed in the distribution of other constitutions between the two groups (Table 3, Supplementary Figure 8). These findings demonstrate remarkable consistency with previous meta-analyses (49, 50), confirming the protective role of the Balanced constitution and the susceptibility patterns associated with Phlegm-dampness, Yin-deficiency, Qi-deficiency, and Blood-stasis constitutions in hypertension.

##### 3.3.2.2 Subgroup analysis

We stratified the 13 included studies by study design type (cross-sectional and case–control) for subgroup analysis (Supplementary Figure 9). Following subgroup analysis, heterogeneity decreased for Yang-deficiency (Supplementary Figure 9C), Dampness-heat (Supplementary Figure 9F), Qi-stagnation (Supplementary Figure 9H), and Special Diathesis constitutions (Supplementary Figure 9I), indicating that study design partially accounted for the high heterogeneity. Although the proportional distribution of TCM constitutions varied across study types, it is evident that the Phlegm-dampness, Balanced, Qi-deficiency, Yin-deficiency, and Yang-deficiency constitutions consistently emerged as the most prevalent in hypertensive population across all design categories. These findings align with the pooled results described above.

#### 3.3.3 Meta-analysis of TCM constitution patterns in AIS with hypertension population

##### 3.3.3.1 Main findings

The meta-analysis included two studies (27, 64) examining TCM constitution in AIS patients with hypertension (Table 2, Supplementary Figure 10). The results indicated that Phlegm-dampness, Qi-deficiency, Yin-deficiency, and Blood-stasis constitutions each accounted for ≥15% of cases, confirming their prevalence in this population. The two studies also compared the distribution of TCM constitution types between AIS patients with hypertension (n = 341) and those without (n = 179). No statistically significant differences were observed in constitutional distribution patterns between groups. This finding, while preliminary due to the limited sample size, suggests the need for further investigation into the role of constitutional factors in AIS patients with hypertension (Table 3, Supplementary Figure 11).

##### 3.3.3.2 Subgroup analysis

Studies were categorized by study design type (cross-sectional or case–control) for subgroup analysis. (Supplementary Figure 12). The limited number of included studies precluded a definitive assessment of whether study design influenced heterogeneity. However, it was noteworthy that the Phlegm-dampness, Blood-stasis, Qi-deficiency, and Yin-deficiency constitutions each accounted for ≥15% of cases in the majority of studies within both design categories. Conversely, the remaining five constitutions consistently each represented <15% across subgroups. This pattern confirms these four constitutions as predominant in the AIS with hypertension population, concordant with the pooled findings.

#### 3.3.4 Meta-analysis of TCM constitution patterns in IS with hypertension population

##### 3.3.4.1 Main findings

A total of 10 studies (27, 64–72) were identified and included in the meta-analysis. The findings showed that Phlegm-dampness, Qi-deficiency, Yin-deficiency, and Blood-stasis constitutions each constituted ≥15% of cases, highlighting their prominence among IS patients with hypertension (Table 2, Supplementary Figure 13). Eight studies investigated the distribution of TCM constitution types in a cohort of 1,955 IS patients, comprising 1,323 with hypertension and 632 without. The results revealed that the Phlegm-dampness constitution (OR = 2.15, 95% CI [1.02, 4.55], *p* = 0.04) was significantly more prevalent in IS patients with hypertension. Conversely, the Yang-deficiency constitution was less common in the hypertensive group (OR = 0.66, 95% CI [0.45, 0.97], *p* = 0.04). These differences were statistically significant (Table 3, Supplementary Figure 14).

##### 3.3.4.2 Subgroup analysis

Studies were classified by study design type (cross-sectional or case–control) for subgroup analysis. (Supplementary Figure 15). After stratification, heterogeneity decreased substantially and was eliminated (I^2^ = 0%) in the case–control subgroups for the following constitutions: Balanced constitution (Supplementary Figure 15A), Yang-deficiency constitution (Supplementary Figure 15C), Dampness-heat constitution (Supplementary Figure 15F), and Blood-stasis constitution (Supplementary Figure 15G). These findings identified study design as a primary driver of the observed heterogeneity. Despite variations in the proportional distribution of constitutions across study types, the Phlegm-dampness, Blood-stasis, Qi-deficiency, and Yin-deficiency constitutions each comprised ≥15% of cases in the majority of studies within both design categories. The remaining five constitutions consistently each accounted for <15% across subgroups. This confirms these four as core TCM constitutions in the IS with hypertension population, consistent with the pooled results.

We further assessed the potential influences of disease severity and stroke subtypes on TCM constitution distribution. No studies addressed associations with disease severity. However, one study (68) examined stroke subtypes, specifically including patients with LAA and/or small artery occlusion (SAO). In this cohort, the predominant constitutions were: Phlegm-dampness, Qi-deficiency, Yin-deficiency, and Blood-stasis—aligning with the meta-analysis results for IS patients with hypertension (Table 2).

#### 3.3.5 Meta-analysis of TCM constitution patterns in hypotensive population

The analysis incorporated three eligible studies (73–75). Significant heterogeneity among studies necessitated the application of random-effects models. The findings revealed that Phlegm-dampness, Qi-deficiency, Yin-deficiency, and Blood-stasis constitutions each accounted for ≥15% of the cases in individuals with hypotension (Table 2, Supplementary Figure 16). Two studies (73, 75) compared TCM constitution types in 635 participants, including 161 with hypotension and 474 with normal BP. The meta-analysis demonstrated that Yin-deficiency (OR = 3.24, 95% CI [1.89, 5.55], *p* < 0.0001) and Qi-deficiency (OR = 3.06, 95% CI [1.41, 6.65], *p* = 0.005) were more prevalent among hypotensive individuals compared to the controls (Table 3, Supplementary Figure 17). All three eligible studies employed cross-sectional designs, precluding subgroup analysis by study type due to homogeneity.

#### 3.3.6 Meta-analysis of TCM constitution patterns in ischemic cerebrovascular diseases with hypoperfusion populations

The meta-analysis incorporated two studies (38, 76) demonstrating that Qi-deficiency, Phlegm-dampness, Damp-heat, and Yin-deficiency constitutions each accounted for at least 15% of cases (Table 2, Supplementary Figure 18). The two studies also evaluated the differences in TCM constitution types between ischemic cerebrovascular patients with hypoperfusion (*n* = 68) and those with normoperfusion (*n* = 56). Due to the limited sample size, the meta-analysis did not identify any significant differences in TCM constitution distribution between the two groups (Table 3, Supplementary Figure 19). Subgroup analysis by study type was inapplicable as all two studies shared identical cross-sectional designs.

### 3.4 Sensitivity analysis

Sensitivity analyses were performed by sequentially excluding each included study. In the meta-analysis of the Special Diathesis constitution among IS with hypertension population, the exclusion of the study by Wu et al. (72) substantially reduced heterogeneity (I^2^ decreased from 53% [*p* = 0.08] to 0% [*p* = 0.78]). However, the pooled estimate was not statistically distinct (pre-exclusion: 2% [0.01, 0.04] vs. post-exclusion: 1% [0.01, 0.02]), confirming the low distribution of Special Diathesis constitution in this population. For the remaining five population subgroups, sequential exclusion of individual studies demonstrated no material alteration in heterogeneity indices or effect sizes, indicating robust primary findings.

### 3.5 Publication bias assessment

**Figure 7:**
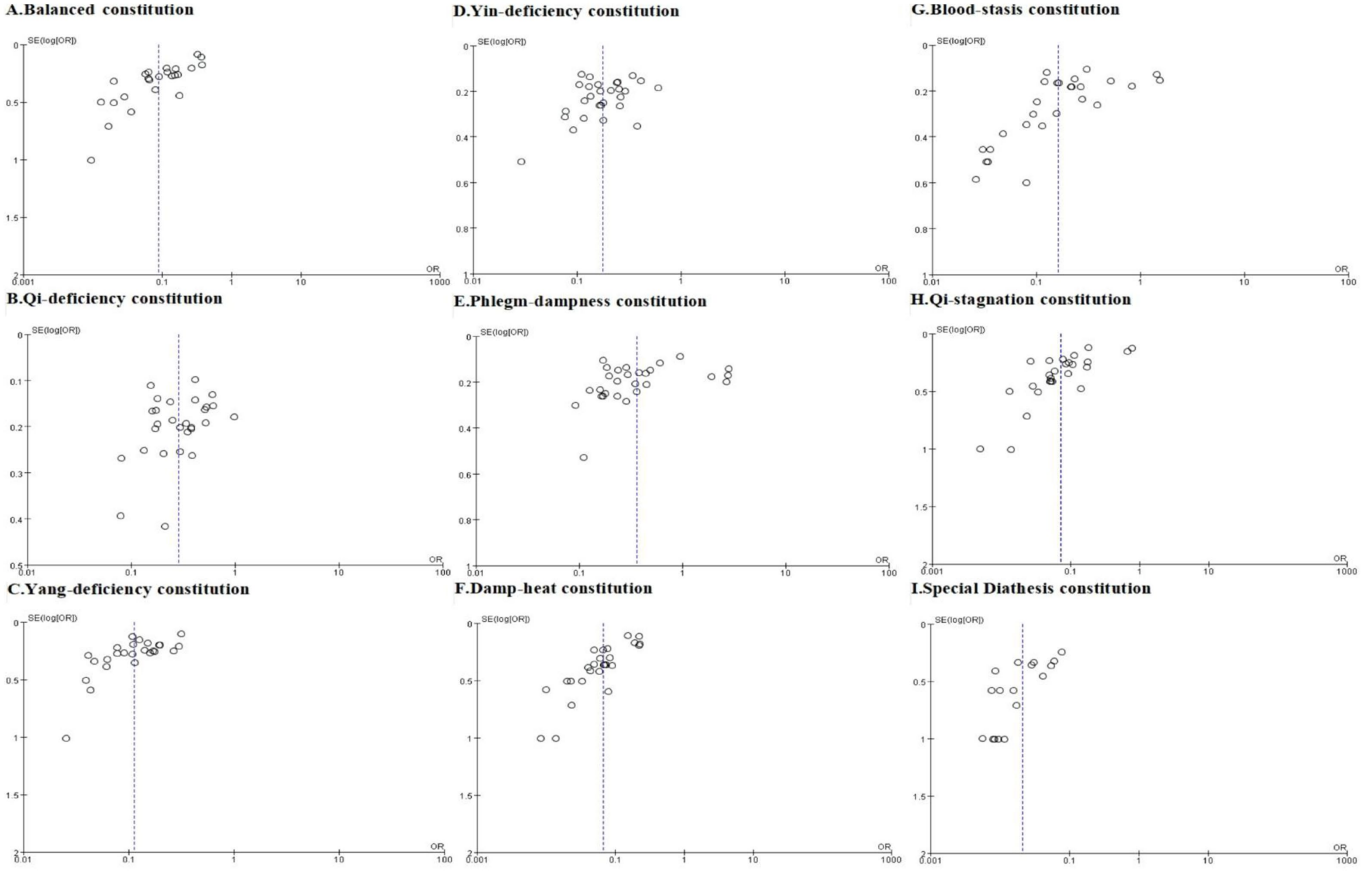
The funnel plots of Balanced constitution **(A)**, Qi-deficiency constitution **(B)**, Yang-deficiency constitution **(C)**, Yin-deficiency constitution **(D)**, Phlegm-dampness constitution **(E)**, Damp-heat constitution **(F)**, Blood-stasis constitution **(G)**, Qi-stagnation constitution **(H)**, and Special Diathesis constitution **(I)** distributions in the AIS population.

**Figure 8:**
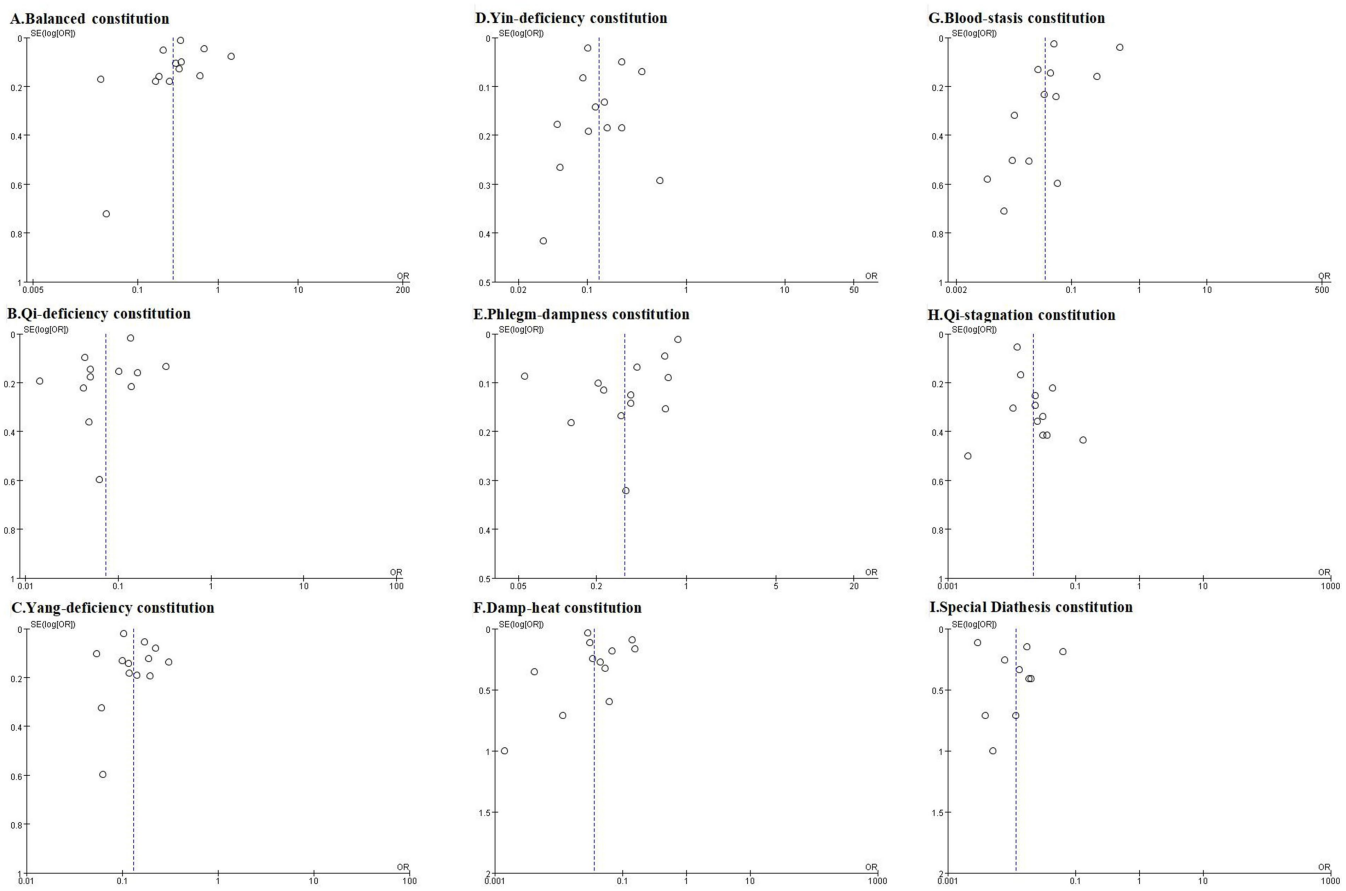
The funnel plots of Balanced constitution **(A)**, Qi-deficiency constitution **(B)**, Yang-deficiency constitution **(C)**, Yin-deficiency constitution **(D)**, Phlegm-dampness constitution **(E)**, Damp-heat constitution **(F)**, Blood-stasis constitution **(G)**, Qi-stagnation constitution **(H)**, and Special Diathesis constitution **(I)** distributions in the hypertensive population.

**Figure 9:**
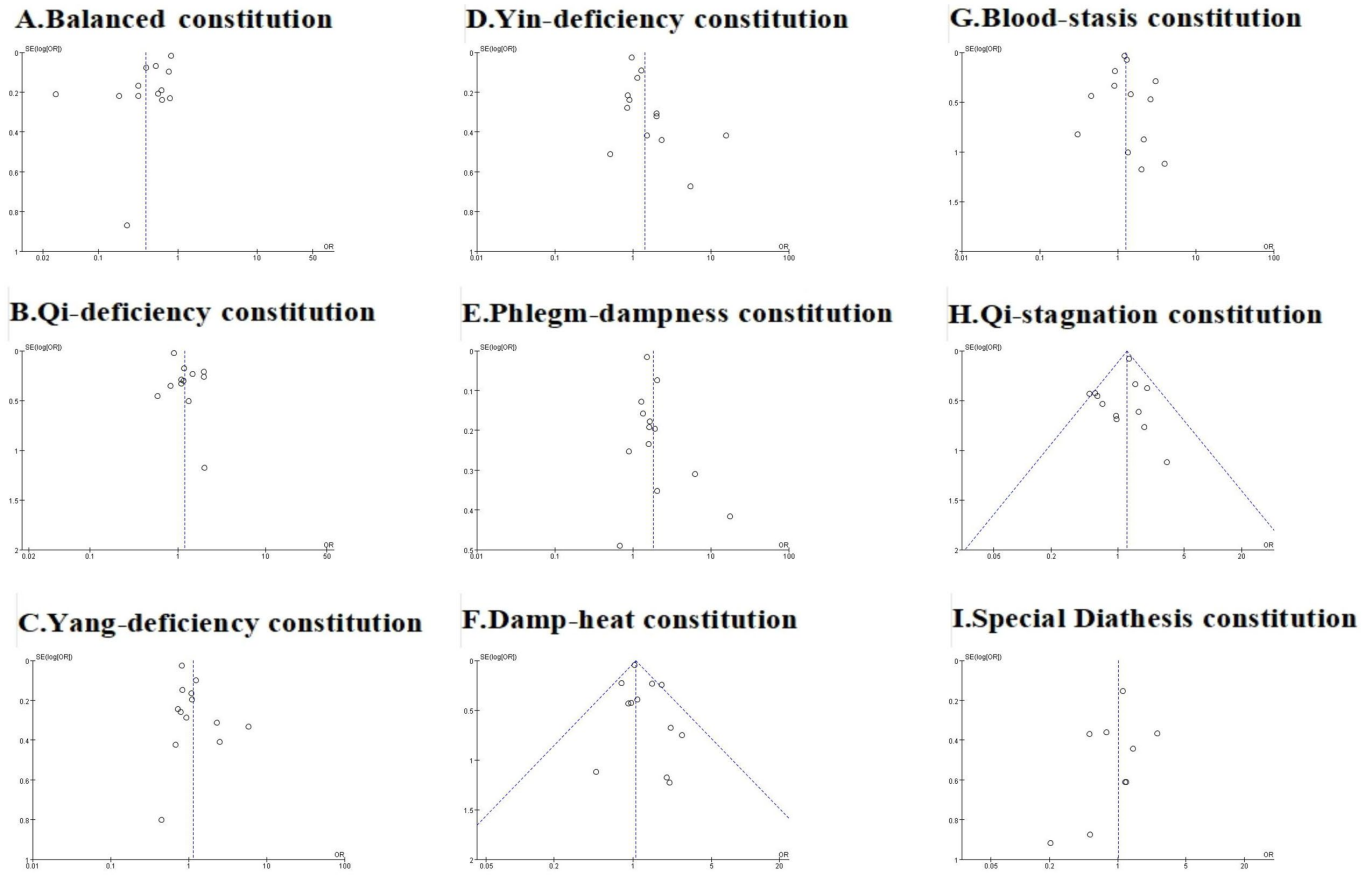
The funnel plots comparing Balanced constitution **(A)**, Qi-deficiency constitution **(B)**, Yang-deficiency constitution **(C)**, Yin-deficiency constitution **(D)**, Phlegm-dampness constitution **(E)**, Damp-heat constitution **(F)**, Blood-stasis constitution **(G)**, Qi-stagnation constitution **(H)**, and Special Diathesis constitution **(I)** distributions between the hypertensive population and the non-hypertensive population.

We assessed publication bias for analyses incorporating more than 10 studies, including meta-analyses of TCM constitution distributions in the AIS population, hypertensive population, and comparative analyses of hypertensive versus non-hypertensive population. Trim-and-fill analysis was performed where publication bias was detected. The overall assessment indicated that while some analyses exhibited publication bias, the original conclusions remained robust after trim-and-fill correction. Funnel plots are presented in Figures 7–9, with Egger’s test, Begg’s test, and trim-and-fill results detailed in Table 4.

**Table 4:** Publication bias assessment with Egger’s test, Begg’s test, and Trim-and-fill analysis.

Population	TCM constitution	Egger’s test		Begg’s test		Trim and fill method	
		Intercept	P	Kendall’s tau	P	Number of imputed studies	Pooled proportion after trim and fill [95% CI], P
AIS Population	Yin-deficiency constitution	−2.207	0.187	−0.159	0.236	N/A	N/A
	Phlegm-dampness constitution	−4.691	0.139	−0.175	0.192	N/A	N/A
	Qi-deficiency constitution	−2.393	0.256	−0.196	0.144	N/A	N/A
	Blood-stasis constitution	−5.549	0.008	−0.345	0.012	0	Data unchanged
	Balanced constitution	−6.089	<0.0001	−0.449	0.002	0	Data unchanged
	Damp-heat constitution	−4.317	<0.0001	−0.366	0.009	0	Data unchanged
	Qi-stagnation constitution	−6.397	<0.0001	−0.280	0.045	0	Data unchanged
	Yang-deficiency constitution	−3.852	<0.0001	−0.372	0.008	0	Data unchanged
	Special Diathesis constitution	−3.032	0.002	−0.250	0.161	0	Data unchanged
Hypertensive Population	Yin-deficiency constitution	2.041	0.449	−0.051	0.807	N/A	N/A
	Phlegm-dampness constitution	−9.853	0.005	−0.026	0.903	2	0.28 [0.14, 0.58], *p* = 0.001
	Qi-deficiency constitution	−3.866	0.074	−0.091	0.681	N/A	N/A
	Blood-stasis constitution	−2.433	0.637	0.205	0.329	N/A	N/A
	Balanced constitution	−0.775	0.812	−0.231	0.272	N/A	N/A
	Damp-heat constitution	1.348	0.592	−0.212	0.337	N/A	N/A
	Qi-stagnation constitution	2.394	0.054	0.182	0.411	N/A	N/A
	Yang-deficiency constitution	1.996	0.294	−0.077	0.714	N/A	N/A
	Special Diathesis constitution	2.170	0.489	−0.111	0.655	N/A	N/A
Hypertensive Population VS Non-hypertensive Population	Yin-deficiency constitution	1.691	0.043	0.231	0.272	0	Data unchanged
	Phlegm-dampness constitution	1.041	0.244	0.231	0.272	N/A	N/A
	Qi-deficiency constitution	1.172	0.030	−0.273	0.217	0	Data unchanged
	Blood-stasis constitution	0.127	0.810	0.077	0.714	N/A	N/A
	Balanced constitution	−4.937	0.012	0	1	4	0.28 [0.15,0.54], P<0.0001
	Damp-heat constitution	0.504	0.204	0.152	0.493	N/A	N/A
	Qi-stagnation constitution	−0.460	0.347	0.061	0.784	N/A	N/A
	Yang-deficiency constitution	1.570	0.059	0	1	N/A	N/A
	Special Diathesis constitution	−0.725	0.440	−0.333	0.211	N/A	N/A

N/A, not applicable; AIS, acute ischemic stroke; TCM, traditional Chinese medicine.

For the AIS population, funnel plots for Yin-deficiency, Phlegm-dampness, and Qi-deficiency constitutions were symmetrical (Figure 7), with both Egger’s and Begg’s tests yielding *p* > 0.05, indicating no significant publication bias. However, funnel plots for Blood-stasis, Balanced, Damp-heat, Qi-stagnation, Yang-deficiency, and Special Diathesis constitutions demonstrated slight asymmetry, with either Egger’s or Begg’s test showing *p* ≤ 0.05, suggesting potential publication bias. Trim-and-fill analysis revealed no studies requiring addition or removal, confirming the robustness of original findings and indicating that publication bias did not substantively impact conclusions.

In the meta-analysis of TCM constitution distribution among hypertensive populations, funnel plots for all nine constitutions appeared generally symmetrical (Figure 8). With the exception of the Phlegm-dampness constitution which demonstrated significant asymmetry on Egger’s test (*p* = 0.005), both Egger’s and Begg’s tests for the remaining eight constitutions yielded non-significant results (*p* > 0.05), indicating no substantial evidence of publication bias. Trim-and-fill analysis for Phlegm-dampness constitution identified two missing studies. After imputation, the pooled prevalence estimate was 28% [0.14, 0.58]—closely aligned with the original effect size in Table 2—thereby reinforcing that Phlegm-dampness is among the most prevalent TCM constitutions in hypertension.

Regarding comparative analyses between hypertensive and non-hypertensive populations, funnel plots demonstrated symmetry for Phlegm-dampness, Blood-stasis, Damp-heat, Qi-stagnation, Yang-deficiency, and Special Diathesis constitutions (Figure 9), with non-significant Egger’s and Begg’s tests (*p* > 0.05), indicating no publication bias. Although Yin-deficiency (Egger’s test: *p* = 0.043; Begg’s test: *p* = 0.272) and Qi-deficiency (Egger’s test: *p* = 0.030; Begg’s test: *p* = 0.217) constitutions showed minor asymmetry, trim-and-fill analysis required no study imputation, confirming minimal bias impact. Balanced constitution exhibited significant funnel plot asymmetry (Egger’s test: *p* = 0.012; Begg’s test: *p* < 0.0001). Trim-and-fill imputation added four studies, yielding a corrected effect size of 0.28 [0.15, 0.54] (*p* < 0.0001). This result remained consistent with the original conclusion that Balanced constitution prevalence is significantly lower in the hypertensive population, affirming its role as a protective factor against hypertension.

## 4 Discussion

### 4.1 Summary of the findings

Approximately 75% of AIS patients present with elevated BP, mediated by neurological injury, autonomic dysfunction, and pre-existing hypertension (3, 4). In addition, acute disruption of cerebral autoregulation after AIS impairs cerebrovascular adaptability to BP fluctuations, exacerbating ischemic tissue vulnerability to both hypoperfusion and hyperperfusion (77). This creates a therapeutic paradox: aggressive early BP reduction may jeopardize penumbral perfusion (78, 79), while sustained hypertension elevates hemorrhagic transformation and reperfusion injury risks, particularly in thrombolysis recipients (80, 81). Large-scale trials (e.g., ENCHANTED, and BEST-II) comparing intensive versus standard BP strategies, have yielded inconclusive results (9–12) due to marked patient heterogeneity. Beyond CA impairment, BP regulation in AIS involves complex interactions among cerebral vasculature pathology, systemic hemodynamics, and endocrine responses, rendering conventional demographic-based stratification inadequate for capturing physiological and pathological variability. This underscores the necessity to transcend numerical BP targets by integrating individualized physiological profiling. Emerging frameworks, including TCM constitutional theory, may enhance precision in predicting antihypertensive responses and tailoring therapeutic strategies.

We therefore systematically investigated TCM constitutional distributions across relevant populations of AIS with hypertension to determine whether these patterns correlate with disease characteristics and therapeutic heterogeneity. Hypertensive and AIS populations share common constitutional patterns, with Phlegm-dampness, Yin-deficiency, Qi-deficiency, and Blood-stasis constitutions being the most prominent. In AIS/IS with hypertensive individuals, the overlap of constitutional profiles, dominated by Phlegm-dampness, Yin-deficiency, Qi-deficiency, and Blood-stasis constitutions, further underscores the shared pathophysiological mechanisms linking hypertension and AIS. These consistent patterns emphasize the fundamental role of the four TCM constitutions in AIS with hypertension, further large-scale studies are warranted to verify these findings. In contrast, the hypotensive population and those with ischemic cerebrovascular hypoperfusion display a unique constitutional distribution. While the Phlegm-dampness, Qi-deficiency, and Yin-deficiency constitutions remain prominent, the Yin-deficiency and Qi-deficiency constitutions are significantly more prevalent compared to normotensive individuals. This distinct constitutional divergence from hypertension and AIS profiles suggests a pathophysiological propensity toward hemodynamic instability, particularly manifesting as hypotension or ischemic cerebrovascular hypoperfusion in individuals with Yin-deficiency and Qi-deficiency constitutions.

The majority of included studies demonstrated moderate-to-high methodological quality as evaluated by standardized tools (AHRQ for cross-sectional studies and NOS for cohort/case–control studies). Subgroup analyses demonstrated that study design constituted a key source of heterogeneity; nevertheless, most subgroups consistently revealed that Phlegm-dampness, Blood-stasis, Qi-deficiency, and Yin-deficiency constitutions persisted as the predominant types across design categories. Sensitivity analyses employing leave-one-out cross-validation confirmed the robustness of primary findings. Publication bias assessments via funnel plots, Egger’s test, and Begg’s test indicated generally symmetrical distributions, with minimal evidence of bias for most constitutions. Where publication bias was detected (e.g., for Phlegm-dampness constitution in hypertensive population), trim-and-fill imputation demonstrated a negligible impact on the original effect sizes, reinforcing the robustness of the primary conclusions. Collectively, these analytical approaches substantiated the reliability and consistency of the identified TCM constitutional patterns underlying the proposed framework.

### 4.2 Targeted BP management in AIS with hypertension based on the TCM constitution

Extensive research has confirmed that distinct TCM constitutional types are associated with unique pathophysiological characteristics and prognostic implications, offering a framework for individualized BP management in AIS with hypertension. Patients with the Phlegm-dampness constitution often present with metabolic abnormalities such as hyperlipidemia and obesity, predisposing them to atherosclerosis (82–84) and elevating risks of BP irregularities and stroke. Mechanistic studies indicated intestinal microbiota imbalance (characterized by elevated Firmicutes/Bacteroidetes ratio), chronic inflammation, and metabolic dysregulation in these patients (85–88). These processes promoted vascular endothelial dysfunction (89), contributing to vascular-related diseases like hypertension and AIS. Regarding Blood-stasis constitution, several clinical studies revealed altered hemorheological parameters and heightened thrombogenic potential (37, 90), resulting in microcirculatory dysfunction and elevated vascular stenosis risk. Modern pathological studies further demonstrated that these abnormalities directly promote atherosclerosis, thrombosis, and endothelial dysfunction (91, 92). From a TCM perspective, both constitutions involve impeded circulation of Qi, blood, and fluids, leading to pathogenic accumulation within vessels. Consequently, elevated BP in these individuals may impose excessive mechanical stress on already compromised vasculature, significantly increasing the risk of acute vascular events, including plaque rupture, thrombosis, and recurrent stroke (93, 94). Intensive BP control may be warranted for patients with these constitutions to optimize cerebral hemodynamics and mitigate their inherently elevated stroke risk associated with vascular vulnerability.

Conversely, Qi-deficiency and Yin-deficiency constitutions pose unique challenges for BP regulation due to compromised autonomic function and diminished compensatory capacity. This renders patients highly susceptible to BP fluctuations and hypoperfusion events (38, 74, 76, 95). TCM pathology attributes Qi deficiency to the depletion of “vital energy” (Qi), manifesting as impaired functional activity that undermines blood circulation regulation. Yin deficiency, characterized by insufficient fluid nourishment, compromises vascular tone and perfusion pressure. Modern studies linked Qi-deficiency to cellular energy metabolism disorders (notably mitochondrial dysfunction) (96, 97), which impair adenosine triphosphate (ATP)-dependent vascular autoregulation and repair mechanisms. Yin deficiency was associated with chronic inflammation and oxidative stress (98, 99), exacerbating endothelial dysfunction and reducing vascular elasticity and reserve (100). These impairments weaken compensatory responses to BP fluctuations, increasing vulnerability to hypoperfusion, especially in critical organs like the brain. Consequently, a conservative BP management strategy may be more appropriate for them to avoid iatrogenic hypoperfusion while managing risks from sustained hypertension.

Taken together, these findings support a hypothesis for TCM constitution-targeted BP management in AIS patients. Patients with Phlegm-dampness or Blood-stasis constitutions, characterized by vascular stenosis, thrombosis propensity, and metabolic dysregulation, likely benefit from intensive BP control to reduce stroke recurrence risk (101). In contrast, those with Qi-deficiency or Yin-deficiency constitutions exhibit hemodynamic fragility and diminished vascular reserve, suggesting that more conservative BP management strategies could potentially be appropriate to help reduce the risk of hypoperfusion complications.

### 4.3 Challenges and opportunities for TCM constitution-based BP management

#### 4.3.1 Limitations

The present meta-analysis provides novel insights into TCM constitution-based BP management in AIS. However, several limitations warrant consideration. Firstly, all studies originated from Chinese populations, potentially introducing cultural and regional biases that limit extrapolation to diverse ethnic groups. Secondly, the limited sample size, particularly for AIS patients with hypertension, underscores the need for larger, multicenter cohorts to validate these preliminary associations. Finally, many studies did not report data on associations between relevant factors (such as stroke subtypes) and TCM constitution distributions. This lack of data precluded subgroup analyses, thereby limiting our ability to perform more detailed characterization of population-specific constitutional profiles.

#### 4.3.2 Clinical implications

The integration of TCM constitutional theory into AIS care might introduce a paradigm shift toward personalized BP management. By categorizing AIS patients into distinct constitutional profiles, such as Phlegm-dampness, or Qi-deficiency, clinicians might better predict hemodynamic vulnerabilities and therefore tailor different therapeutic strategies. This approach not only refines acute care protocols by aligning BP-lowering strategies but also enhances long-term secondary prevention through targeted interventions, such as lifestyle modifications. While rooted in TCM, this framework aligns with global precision medicine initiatives, offering a low-cost, non-invasive tool adaptable to diverse populations.

#### 4.3.3 Future directions

To translate these findings into practice, the following priorities must be addressed: (1) Standardization: Developing internationally recognized criteria for TCM constitution classification, supported by objective biomarkers (e.g., inflammatory markers, hemodynamic parameters) to reduce subjectivity. (2) Mechanistic Exploration: Investigating the biological underpinnings of constitutional types using omics technologies (genomics, proteomics) to elucidate their interactions with cerebral autoregulation and BP homeostasis. (3) Interventional Trials: Designing adaptive trials comparing constitution-guided BP management versus standard care, with endpoints including functional outcomes, complication rates, and quality of life. Subgroup analyses should explicitly incorporate key clinical variables such as disease severity and stroke subtype to assess their association with the TCM constitution and evaluate the differential efficacy of intensive versus conservative strategies across constitutional profiles. (4) Global Collaboration: Establishing multinational registries to evaluate the cross-cultural applicability of TCM constitutional theory and address ethnic/geographic variability in stroke pathophysiology. (5) Technology Integration: Leveraging artificial intelligence to automate constitutional assessments using clinical, imaging, and laboratory data, thereby enhancing scalability and precision.

## 5 Conclusion

This study proposes a targeted BP management hypothesis for AIS patients by integrating TCM constitution theory with modern medical practices. Through systematic meta-analyses, we identified distinct constitutional patterns and their specific therapeutic requirements in AIS patients with hypertension. Our findings reveal that Phlegm-dampness and Blood-stasis constitutions may benefit from intensive BP management, while Qi-deficiency and Yin-deficiency constitutions may require conservative approaches. By addressing patient-specific needs, the study highlights the potential of integrating the TCM constitution and modern medicine to optimize recovery, lower stroke recurrence risk, and advance personalized healthcare.

## Data Availability

The original contributions presented in the study are included in the article/Supplementary material, further inquiries can be directed to the corresponding author.

## Glossary

AHRQ: Agency for Healthcare Research and Quality
AIS: Acute Ischemic Stroke
ATP: Adenosine Triphosphate
BP: Blood Pressure
CACM: China Association of Chinese Medicine
CBM: China Biology Medicine
CI: Confidence Interval
CNKI: China National Knowledge Infrastructure
IS: Ischemic Stroke
LAA: Large Artery Atherosclerosis
MeSH: Medical Subject Headings
NIHSS: National Institutes of Health Stroke Scale
NOS: Newcastle-Ottawa Scale
NR: Not Reported
N/A: Not Applicable
OR: Odds Ratio
PRISMA: Preferred Reporting Items for Systematic Reviews and Meta-Analyses
PROSPERO: Prospective Register of Systematic Reviews
SAO: Small Artery Occlusion
TCM: Traditional Chinese Medicine
