# Supplementary material for: Bridging tradition and innovation: a constitution-guided framework for personalized blood pressure management in acute ischemic stroke
Source: Front Med (Lausanne). 2025 Jul 2;12:1602274. doi: 10.3389/fmed.2025.1602274 (PMC12263929; doi:10.3389/fmed.2025.1602274)
Supplement: Supplementary file 1 [file Table_1.docx]

**Supplementary Table 1. The search strategy of PubMed on TCM Constitution in AIS Patients.**

| **Number** | **Search terms** |
| --- | --- |
| #1 | acute ischemic stroke [MeSH] |
| #2 | stroke [Title] OR ischemic stroke [Title] OR cerebral infarction [Title] OR cerebral embolism [Title] OR infarction [Title] OR ischemic cerebrovascular disease [Title] OR cerebral thrombosis [Title] OR lacunar infarction [Title] OR cerebral apoplexy [Title] OR cerebral vascular accident [Title] OR cva [Title] OR apoplexy [Title] OR infarction of the brain [Title] |
| #3 | #1 OR #2 |
| #4 | constitution [Title] |
| #5 | acute [Title/Abstract] |
| #6 | traditional Chinese medicine [All Fields] |
| #7 | #3 AND #4 AND #5 AND #6 |

**Supplementary Table 2. The search strategy of PubMed on TCM Constitution in Hypertensive Patients.**

| **Number** | **Search terms** |
| --- | --- |
| #1 | hypertension [MeSH] |
| #2 | hypertension [Title] OR high blood pressure [Title] OR hypertensive [Title] |
| #3 | #1 OR #2 |
| #4 | constitution [Title] |
| #5 | traditional Chinese medicine [All Fields] |
| #6 | case-control study [Publication Type] OR cohort study [Publication Type] OR cross-sectional study [Publication Type] |
| #7 | #3 AND #4 AND #5 AND #6 |

**Supplementary Table 3. The search strategy of PubMed on TCM Constitution in Hypotensive Patients.**

| **Number** | **Search terms** |
| --- | --- |
| #1 | hypotension [MeSH] |
| #2 | hypotension [Title] OR low blood pressure [Title] OR hypotensive [Title] |
| #3 | #1 OR #2 |
| #4 | constitution [Title] |
| #5 | traditional Chinese medicine [All Fields] |
| #6 | #3 AND #4 AND #5 |

**Supplementary Table 4. The search strategy of PubMed on TCM Constitution in AIS Patients with Hypertension.**

| **Number** | **Search terms** |
| --- | --- |
| #1 | acute ischemic stroke [MeSH] |
| #2 | stroke [Title] OR ischemic stroke [Title] OR cerebral infarction [Title] OR cerebral embolism [Title] OR infarction [Title] OR ischemic cerebrovascular disease [Title] OR cerebral thrombosis [Title] OR lacunar infarction [Title] OR cerebral apoplexy [Title] OR cerebral vascular accident [Title] OR cva [Title] OR apoplexy [Title] OR infarction of the brain [Title] |
| #3 | hypertension [Title/Abstract] OR high blood pressure [Title/Abstract] OR hypertensive [Title/Abstract] |
| #4 | constitution [Title] |
| #5 | acute [Title/Abstract] |
| #6 | traditional Chinese medicine [All Fields] |
| #7 | #1 OR #2 |
| #8 | #7 AND #3 AND #4 AND #5 AND #6 |

**Supplementary Table 5. The search strategy of PubMed on TCM Constitution in IS Patients with Hypertension.**

| **Number** | **Search terms** |
| --- | --- |
| #1 | ischemic stroke [MeSH] |
| #2 | stroke [Title] OR ischemic stroke [Title] OR cerebral infarction [Title] OR cerebral embolism [Title] OR infarction [Title] OR ischemic cerebrovascular disease [Title] OR cerebral thrombosis [Title] OR lacunar infarction [Title] OR cerebral apoplexy [Title] OR cerebral vascular accident [Title] OR cva [Title] OR apoplexy [Title] OR infarction of the brain [Title] |
| #3 | hypertension [Title/Abstract] OR high blood pressure [Title/Abstract] OR hypertensive [Title/Abstract] |
| #4 | constitution [Title] |
| #5 | traditional Chinese medicine [All Fields] |
| #6 | #1 OR #2 |
| #7 | #6 AND #3 AND #4 AND #5 |

**Supplementary Table 6. The search strategy of PubMed on TCM Constitution in Ischemic Cerebrovascular Patients with Hypoperfusion.**

| **Number** | **Search terms** |
| --- | --- |
| #1 | ischemic stroke [MeSH] |
| #2 | stroke [Title] OR ischemic stroke [Title] OR cerebral infarction [Title] OR cerebral embolism [Title] OR infarction [Title] OR ischemic cerebrovascular disease [Title] OR cerebral thrombosis [Title] OR lacunar infarction [Title] OR cerebral apoplexy [Title] OR cerebral vascular accident [Title] OR cva [Title] OR apoplexy [Title] OR infarction of the brain [Title] |
| #3 | hypoperfusion [Title/Abstract] OR low blood pressure [Title/Abstract] OR hypotension [Title/Abstract] OR hypotensive[Title/Abstract] |
| #4 | constitution [Title] |
| #5 | traditional Chinese medicine [All Fields] |
| #6 | #1 OR #2 |
| #7 | #6 AND #3 AND #4 AND #5 |

**Supplementary Table 7. Results of the AHRQ scale assessment for cross-sectional studies.**

| **Study ID** | **Q1** | **Q2** | **Q3** | **Q4** | **Q5** | **Q6** | **Q7** | **Q8** | **Q9** | **Q10** | **Q11** | **Total points** | **Quality classification** |
| --- | --- | --- | --- | --- | --- | --- | --- | --- | --- | --- | --- | --- | --- |
| Beibei Li 2021 | 1 | 1 | 1 | 0 | 0 | 0 | 1 | 1 | 1 | 0 | N/A | 6 | moderate quality |
| Fang Liu 2017 | 1 | 1 | 1 | 0 | 0 | 0 | 1 | 0 | 1 | 0 | N/A | 5 | moderate quality |
| Fazhi Yang 2023 | 1 | 1 | 1 | 0 | 0 | 1 | 1 | 1 | 1 | 0 | N/A | 7 | high quality |
| Guofang Yang 2009 | 1 | 1 | 1 | 0 | 0 | 0 | 1 | 0 | 1 | 0 | N/A | 5 | moderate quality |
| Jiajing Yuan 2021 | 1 | 1 | 1 | 0 | 0 | 1 | 1 | 1 | 1 | 0 | N/A | 7 | high quality |
| Ling Huang 2019 | 1 | 1 | 1 | 0 | 0 | 0 | 1 | 0 | 1 | 0 | N/A | 5 | moderate quality |
| Liqin Li 2018 | 1 | 1 | 1 | 0 | 0 | 0 | 1 | 0 | 1 | 0 | N/A | 5 | moderate quality |
| Qun Wang 2012 | 1 | 1 | 1 | 0 | 0 | 0 | 1 | 0 | 1 | 0 | N/A | 5 | moderate quality |
| Ran Li 2023 | 1 | 1 | 1 | 0 | 0 | 0 | 1 | 0 | 1 | 0 | N/A | 5 | moderate quality |
| Sainan Cui 2018 | 1 | 1 | 1 | 0 | 0 | 0 | 1 | 0 | 1 | 0 | N/A | 5 | moderate quality |
| Shuhan Shi 2022 | 1 | 1 | 1 | 0 | 0 | 0 | 1 | 1 | 1 | 0 | N/A | 6 | moderate quality |
| Xinxiao Fu 2018 | 1 | 1 | 1 | 0 | 0 | 0 | 1 | 0 | 1 | 0 | N/A | 5 | moderate quality |
| Yan Yan 2021 | 1 | 1 | 1 | 0 | 0 | 0 | 1 | 0 | 1 | 0 | N/A | 5 | moderate quality |
| Yingxiang Liang 2018 | 1 | 1 | 1 | 0 | 0 | 0 | 1 | 0 | 1 | 0 | N/A | 5 | moderate quality |
| Yinping Xu 2015 | 1 | 1 | 1 | 0 | 0 | 1 | 1 | 0 | 1 | 0 | N/A | 6 | moderate quality |
| Yumei Li 2015 | 1 | 1 | 1 | 0 | 0 | 0 | 1 | 0 | 1 | 0 | N/A | 5 | moderate quality |
| Yun Dong 2019 | 1 | 1 | 1 | 0 | 0 | 0 | 1 | 1 | 1 | 0 | N/A | 6 | moderate quality |
| Yunyun Zhang 2014 | 1 | 1 | 1 | 0 | 0 | 0 | 1 | 0 | 1 | 0 | N/A | 5 | moderate quality |
| Zhiwen Li 2017 | 1 | 1 | 1 | 0 | 0 | 0 | 1 | 1 | 1 | 0 | N/A | 6 | moderate quality |
| Zhu Li 2016 | 1 | 1 | 1 | 0 | 0 | 0 | 1 | 0 | 1 | 0 | N/A | 5 | moderate quality |
| Han Zhou 2020 | 1 | 1 | 1 | 0 | 0 | 0 | 1 | 0 | 1 | 0 | N/A | 5 | moderate quality |
| Xuemei Qiu 2021 | 1 | 1 | 1 | 0 | 0 | 0 | 1 | 0 | 1 | 0 | N/A | 5 | moderate quality |
| Birong Lin 2019 | 1 | 1 | 1 | 0 | 0 | 1 | 1 | 1 | 1 | 1 | N/A | 8 | high quality |
| Fan Yang 2024 | 1 | 1 | 1 | 0 | 0 | 1 | 1 | 1 | 1 | 0 | N/A | 7 | high quality |
| He Li 2022 | 1 | 1 | 1 | 0 | 0 | 1 | 1 | 1 | 1 | 0 | N/A | 7 | high quality |
| Jian Xiao 2023 | 1 | 1 | 1 | 0 | 0 | 0 | 1 | 1 | 1 | 0 | N/A | 6 | moderate quality |
| Lihua Chen 2023 | 1 | 1 | 1 | 0 | 0 | 1 | 1 | 0 | 1 | 0 | N/A | 6 | moderate quality |
| Xing Zhang 2022 | 1 | 1 | 1 | 0 | 0 | 1 | 1 | 1 | 1 | 1 | N/A | 8 | high quality |
| Xixi Wang 2023 | 1 | 0 | 1 | 0 | 0 | 0 | 0 | 0 | 1 | 0 | N/A | 3 | low quality |
| Qi Liu 2018 | 1 | 1 | 1 | 0 | 0 | 1 | 1 | 1 | 1 | 0 | N/A | 7 | high quality |
| Xiangdong Wang 2015 | 1 | 1 | 1 | 0 | 0 | 0 | 1 | 0 | 1 | 0 | N/A | 5 | moderate quality |
| Ziqu Zhang 2021 | 1 | 1 | 1 | 1 | 0 | 0 | 1 | 0 | 1 | 0 | N/A | 6 | moderate quality |
| Hongjuan Yu 2018 | 1 | 1 | 1 | 0 | 0 | 0 | 1 | 0 | 1 | 0 | N/A | 5 | moderate quality |
| Huabiao Wang 2018 | 1 | 1 | 1 | 0 | 0 | 1 | 1 | 1 | 1 | 1 | N/A | 8 | high quality |
| Kewei Li 2019 | 1 | 1 | 1 | 0 | 0 | 0 | 1 | 1 | 0 | 0 | N/A | 5 | moderate quality |
| Qiang Li 2016 | 1 | 1 | 1 | 0 | 0 | 0 | 1 | 0 | 1 | 1 | N/A | 6 | moderate quality |
| Shuhui Han 2012 | 1 | 1 | 1 | 0 | 0 | 0 | 1 | 0 | 0 | 1 | N/A | 5 | moderate quality |
| Tiantian Song 2023 | 1 | 1 | 1 | 0 | 0 | 1 | 1 | 1 | 1 | 0 | N/A | 7 | high quality |
| Xiaolin Wu 2018 | 1 | 1 | 1 | 1 | 0 | 1 | 1 | 1 | 1 | 0 | N/A | 8 | high quality |
| Ziye Chen 2023 | 1 | 1 | 1 | 0 | 0 | 1 | 1 | 1 | 1 | 0 | N/A | 7 | high quality |
| Q, question. | | | | | | | | | | | | | |

**Supplementary Table 8. Results of the NOS scale assessment for cohort and case-control studies.**

| **Study ID** | **Research type** | **V1** | **V2** | **V3** | **V4** | **V5** | **V6** | **V7** | **V8** | **Total points** | **Quality classification** |
| --- | --- | --- | --- | --- | --- | --- | --- | --- | --- | --- | --- |
| Bingfang Cai 2020 | Cohort study | 1 | 0 | 1 | 1 | 2 | 1 | 0 | 0 | 6 | moderate quality |
| Haimei Zhang 2010 | Cohort study | 1 | 0 | 1 | 1 | 2 | 1 | 1 | 1 | 8 | high quality |
| Xia Liu 2016 | Cohort study | 1 | 0 | 1 | 1 | 2 | 1 | 0 | 0 | 6 | moderate quality |
| Xiaomin Zhen 2015 | Case-control study | 1 | 1 | 1 | 1 | 2 | 1 | 0 | 0 | 7 | high quality |
| Yingping Gao 2022 | Cohort study | 1 | 0 | 1 | 1 | 2 | 1 | 1 | 1 | 8 | high quality |
| Zhiqiang Zou 2020 | Case-control study | 1 | 1 | 1 | 1 | 2 | 1 | 0 | 0 | 7 | high quality |
| Jiefeng Lin 2022 | Case-control study | 1 | 1 | 1 | 1 | 2 | 1 | 0 | 0 | 7 | high quality |
| Mina Zhang 2019 | Case-control study | 1 | 1 | 1 | 1 | 2 | 1 | 0 | 0 | 7 | high quality |
| Shiyuan Jin 2019 | Case-control study | 1 | 1 | 1 | 1 | 2 | 1 | 0 | 0 | 7 | high quality |
| Shunhua Fan 2020 | Case-control study | 1 | 1 | 1 | 1 | 2 | 1 | 0 | 0 | 7 | high quality |
| Yajing Gao 2022 | Case-control study | 0 | 1 | 1 | 1 | 1 | 1 | 0 | 0 | 5 | moderate quality |
| Yuhua Li 2019 | Case-control study | 1 | 1 | 1 | 1 | 2 | 1 | 0 | 0 | 7 | high quality |
| Jing Li 2020 | Case-control study | 1 | 1 | 1 | 1 | 2 | 1 | 0 | 0 | 7 | high quality |
| Kaixin Wang 2012 | Case-control study | 0 | 1 | 1 | 1 | 1 | 1 | 0 | 0 | 5 | moderate quality |
| V,variable. | | | | | | | | | | | |

Records confirmed through 9 databases searching (n=157): CNKI (n=56), VIP (n=23), Wanfang (n=52), CBM (n=26), PubMed (n=0), The Cochrane Library (n=0), Web of Science (n=0), Scopus (n=0), and ScienceDirect (n=0).

Identification

Records after duplicates eliminated (n=83)

Records excluded (n=43)

Not related to AIS (n=24);

Not related to TCM constitution (n=4);

Not related to human (n=2);

Unable to obtain data on TCM constitution in AIS (n=13).

Screening

Full-text articles evaluated for eligibility (n=40)

Eligibility

Full-text excluded (n=12)

Data incomplete or full text unavailable (n=5);

Duplicate reports on the same population (n=3)；

Not based on the referenced standard for constitution classification (n=4).

Included

Studies included in meta-analysis (n=28)

Additional records identified through other sources (n=0).

**Supplementary Figure 1. The Literature Screening Flowchart for Studies on TCM Constitution in AIS Patients.**

Records confirmed through 9 databases searching (n=306): CNKI (n=103), VIP (n=60), Wanfang (n=88), CBM (n=48), PubMed (n=2), The Cochrane Library (n=1), Web of Science (n=1), Scopus (n=2), and ScienceDirect (n=1).

Identification

Records after duplicates eliminated (n=159)

Records excluded (n=132)

Not hypertensive populations (n=14);

Not related to TCM constitution (n=3);

Studies focusing on a single constitution type (n=10);

Not a case-control study, cohort study, or cross-sectional study with a control group (n=90);

Hypertension combined with other diseases or special types of hypertension (n=15).

Screening

Full-text articles evaluated for eligibility (n=27)

Eligibility

Full-text excluded (n=14)

Inappropriate grouping or comparison (n=5)；

Data incomplete or full text unavailable (n=2);

Not based on the referenced standard for constitution classification (n=7).

Included

Studies included in meta-analysis (n=13)

Additional records identified through other sources (n=0).

**Supplementary Figure 2. The Literature Screening Flowchart for Studies on TCM Constitution in Hypertensive Patients.**

Records confirmed through 9 databases searching (n=94): CNKI (n=30), VIP (n=25), Wanfang (n=30), CBM (n=9), PubMed (n=0), The Cochrane Library (n=0), Web of Science (n=0), Scopus (n=0), and ScienceDirect (n=0).

Identification

Records after duplicates eliminated (n=45)

Records excluded (n=41)

Not hypotension populations (n=4);

Not related to TCM constitution (n=33);

Hypotension combined with other diseases or special types of hypotension (n=3);

Studies focusing on a single constitution type (n=1).

Screening

Full-text articles evaluated for eligibility (n=4)

Eligibility

Full-text excluded (n=1)

Not based on the referenced standard for constitution classification (n=1).

Included

Studies included in meta-analysis (n=3)

Additional records identified through other sources (n=0).

**Supplementary Figure 3. The Literature Screening Flowchart for Studies on TCM Constitution in Hypotensive Patients.**

Records confirmed through 9 databases searching (n=85): CNKI (n=38), VIP (n=2), Wanfang (n=43), CBM (n=2), PubMed (n=0), The Cochrane Library (n=0), Web of Science (n=0), Scopus (n=0), and ScienceDirect (n=0).

Identification

Records after duplicates eliminated (n=68)

Records excluded (n=44)

Not related to AIS or hypertension (n=32);

Not related to TCM constitution (n=10);

Studies focusing on a single constitution type (n=2).

Screening

Full-text articles evaluated for eligibility (n=24)

Eligibility

Full-text excluded (n=22)

Unable to obtain TCM constitution data for AIS with hypertension (n=18);

Data incomplete or full text unavailable (n=2);

Duplicate reports on the same population (n=2).

Included

Studies included in meta-analysis (n=2)

Additional records identified through other sources (n=0).

**Supplementary Figure 4. The Literature Screening Flowchart for Studies on TCM Constitution in AIS Patients with Hypertension.**

Records confirmed through 9 databases searching (n=152): CNKI (n=41), VIP (n=22), Wanfang (n=69), CBM (n=20), PubMed (n=0), The Cochrane Library (n=0), Web of Science (n=0), Scopus (n=0), and ScienceDirect (n=0).

Identification

Records after duplicates eliminated (n=88)

Records excluded (n=39)

Not related to IS or hypertension (n=29);

Not related to TCM constitution (n=5);

Ischemic stroke in special types or conditions (n=3);

Studies focusing on a single constitution type (n=2).

Screening

Full-text articles evaluated for eligibility (n=49)

Eligibility

Full-text excluded (n=39)

Unable to obtain TCM constitution data for IS with hypertension (n=31);

Data incomplete or full text unavailable (n=3);

Duplicate reports on the same population (n=2);

Not based on the referenced standard for constitution classification (n=3).

Included

Studies included in meta-analysis (n=10)

Additional records identified through other sources (n=0).

**Supplementary Figure 5. The Literature Screening Flowchart for Studies on TCM Constitution in IS Patients with Hypertension.**

Records confirmed through 9 databases searching (n=26): CNKI (n=5), VIP (n=2), Wanfang (n=17), CBM (n=2), PubMed (n=0), The Cochrane Library (n=0), Web of Science (n=0), Scopus (n=0), and ScienceDirect (n=0).

Identification

Records after duplicates eliminated (n=21)

Records excluded (n=17)

Not related to ischemic cerebrovascular diseases or hypoperfusion (n=10);

Not related to TCM constitution (n=2);

Not related to humans (n=5).

Screening

Full-text articles evaluated for eligibility (n=4)

Eligibility

Full-text excluded (n=2)

Unable to obtain TCM constitution data for ischemic cerebrovascular patients with hypoperfusion (n=2).

Included

Studies included in meta-analysis (n=2)

Additional records identified through other sources (n=0).

**Supplementary Figure 6. The Literature Screening Flowchart for Studies on TCM Constitution in Ischemic Cerebrovascular Patients with Hypoperfusion.**

**
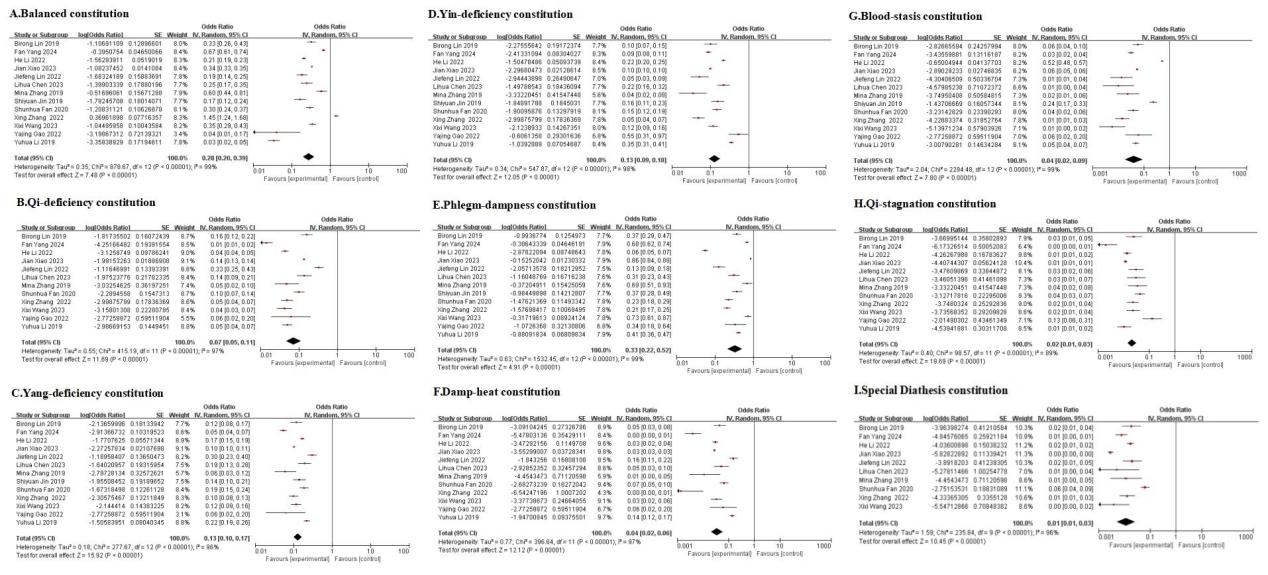
**

**Supplementary Figure 7. **The forest plots of nine TCM** constitution distributions in hypertensive population.**

**
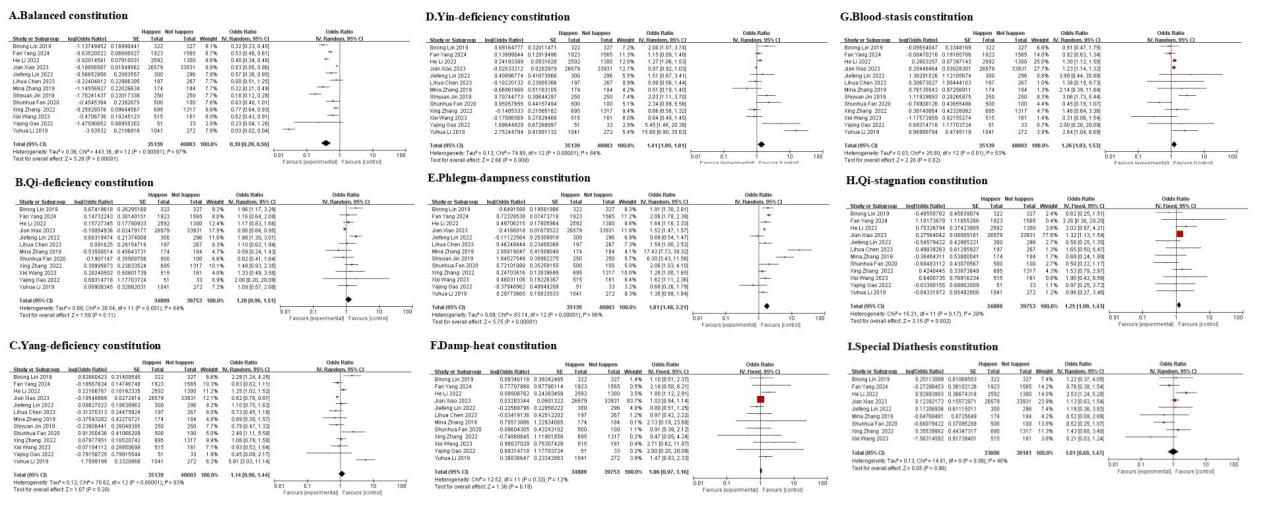
**

**Supplementary Figure 8. **The forest plots** comparing TCM constitution distributions between hypertensive population and non-hypertensive population.**

**
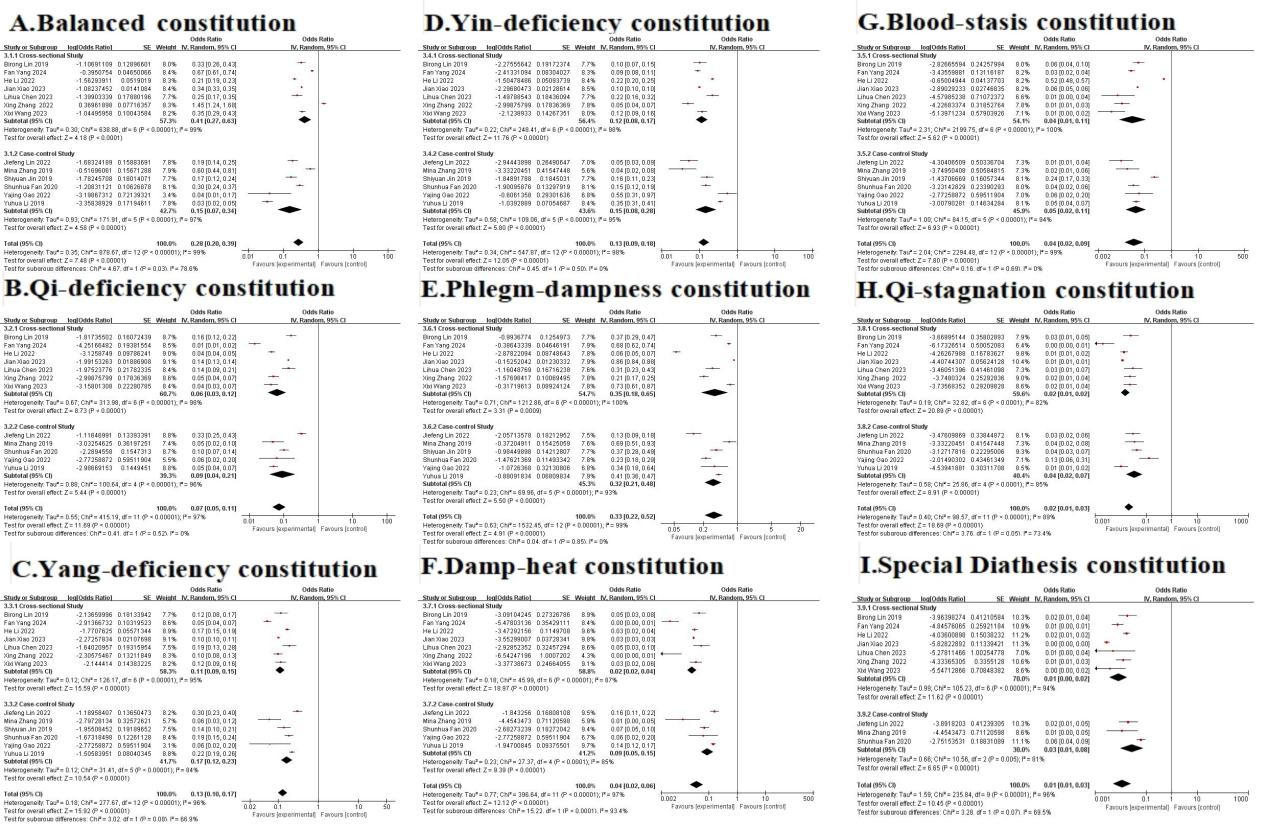
**

#### **Supplementary Figure **9. The forest plot of subgroup analysis for the** hypertensive population **by study design.****

**
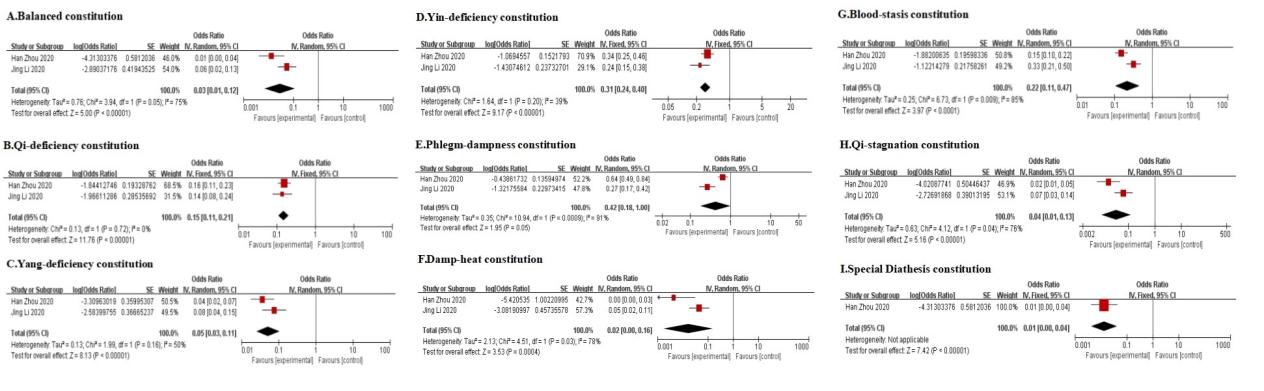
**

**Supplementary Figure 10. **The forest plots of nine TCM** constitution distributions in AIS with hypertension population.**

**
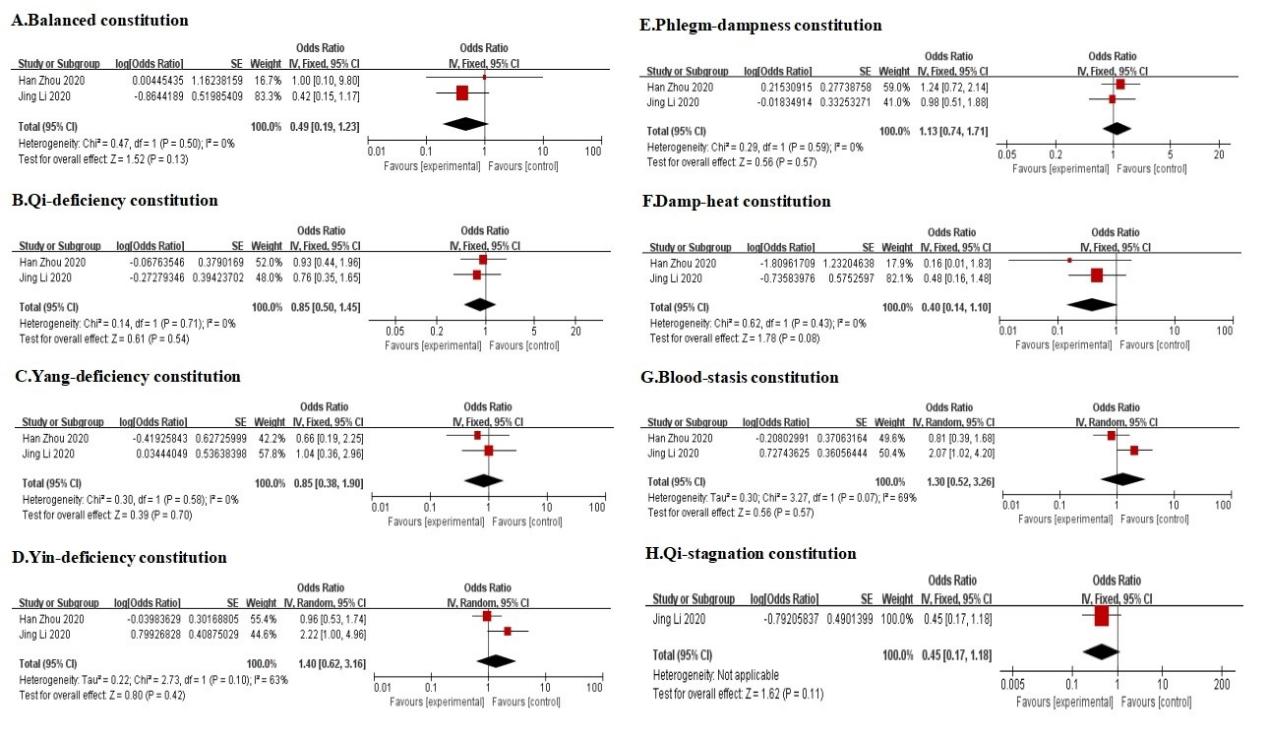
**

**Supplementary Figure 11. **The forest plots** comparing TCM constitution distributions between AIS with hypertension population and AIS without hypertension population.**

**
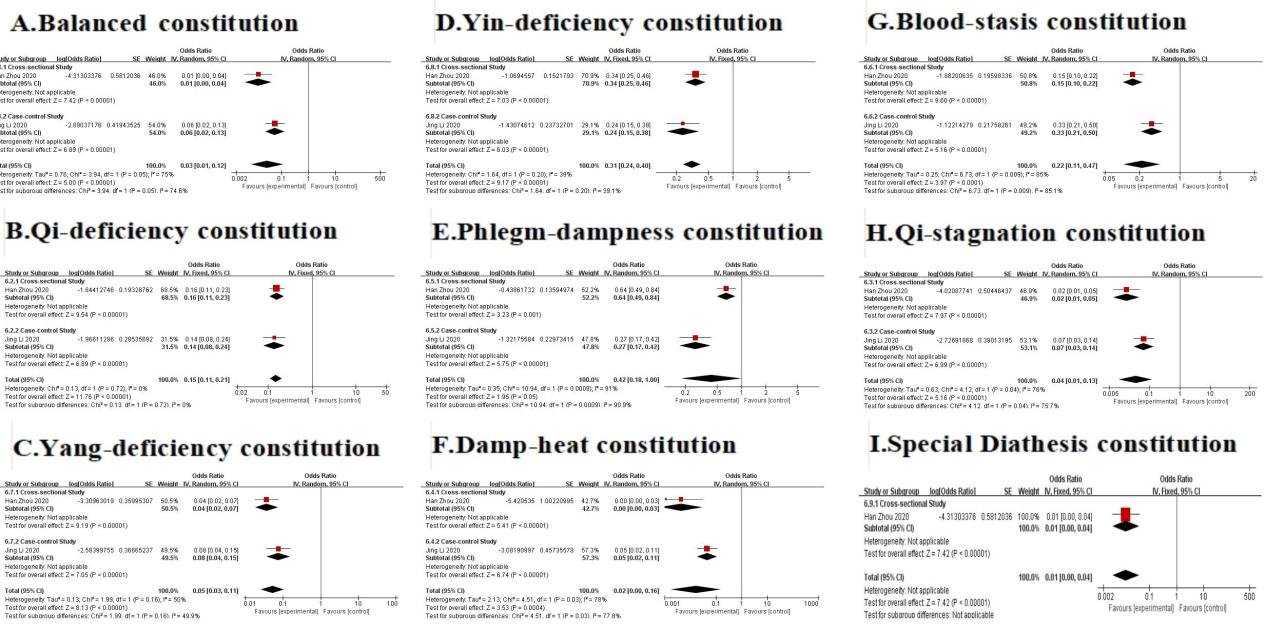
Supplementary Figure **12. The forest plot of subgroup analysis for the** AIS with hypertension **population by study design.****

**
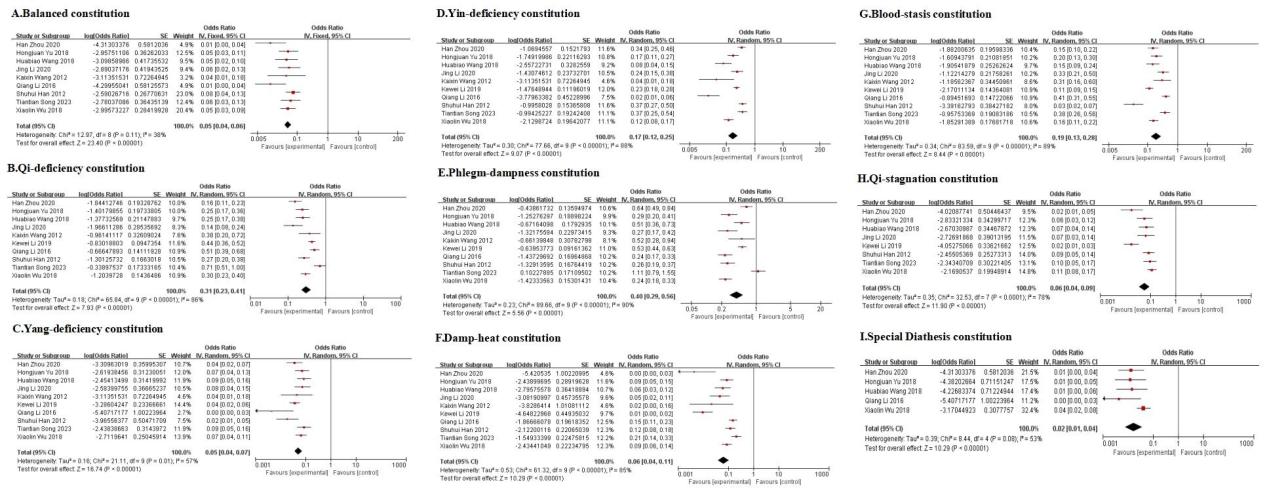
Supplementary Figure 13. **The forest plots of nine TCM** constitution distributions in IS with hypertension population.**

**
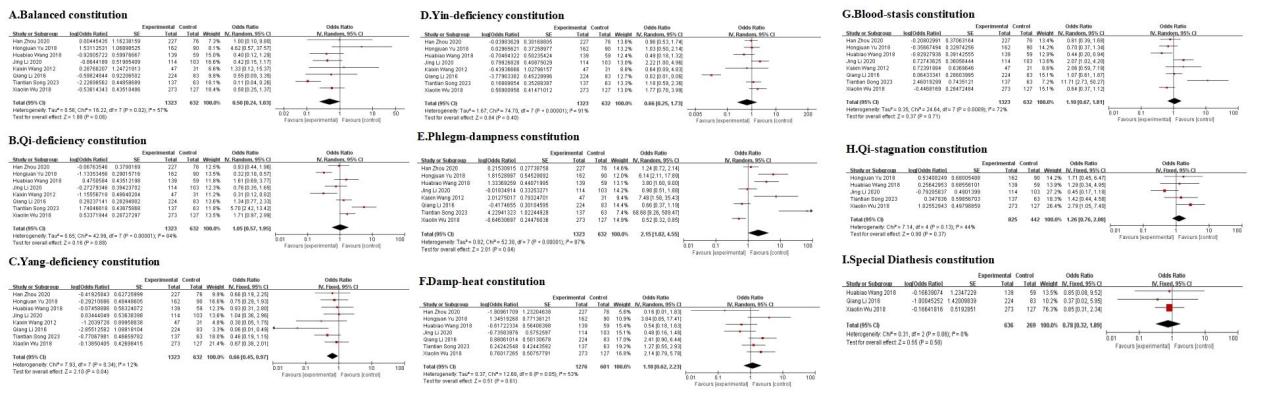
Supplementary Figure 14. **The forest plots** comparing TCM constitution distributions between IS with hypertension population and IS without hypertension population.**

**
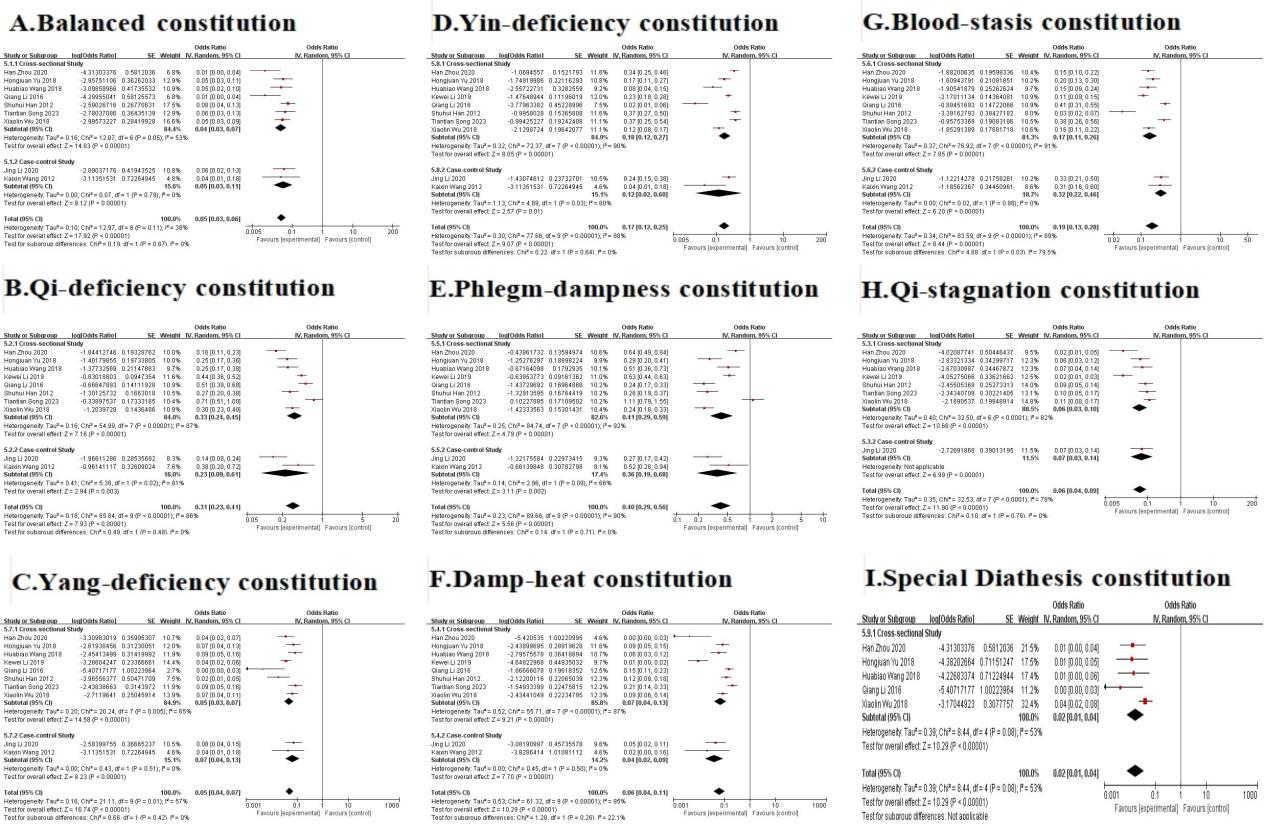
Supplementary Figure **15. The forest plot of subgroup analysis for the** IS with hypertension **population by study design.****

**
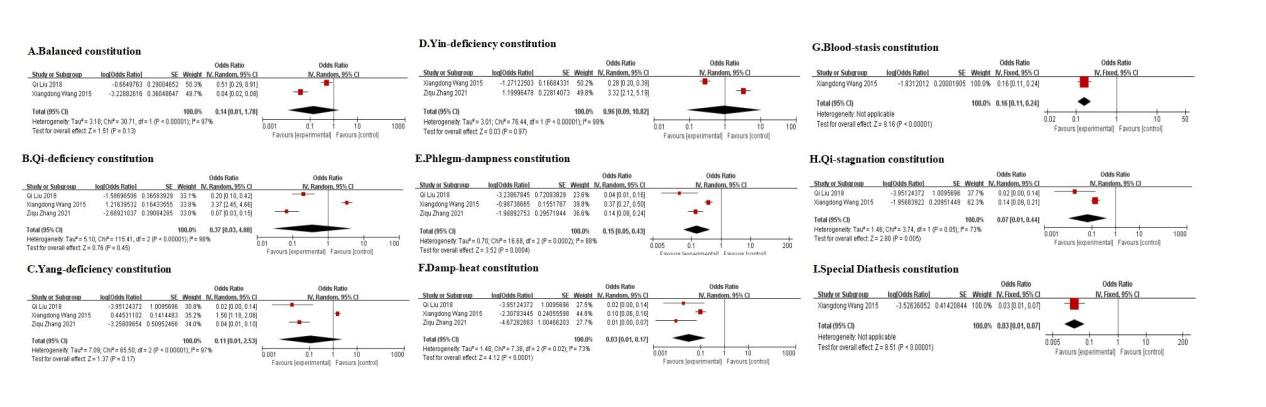
**

**Supplementary Figure 16. **The forest plots of nine TCM** constitution distributions in hypotensive population.**

**
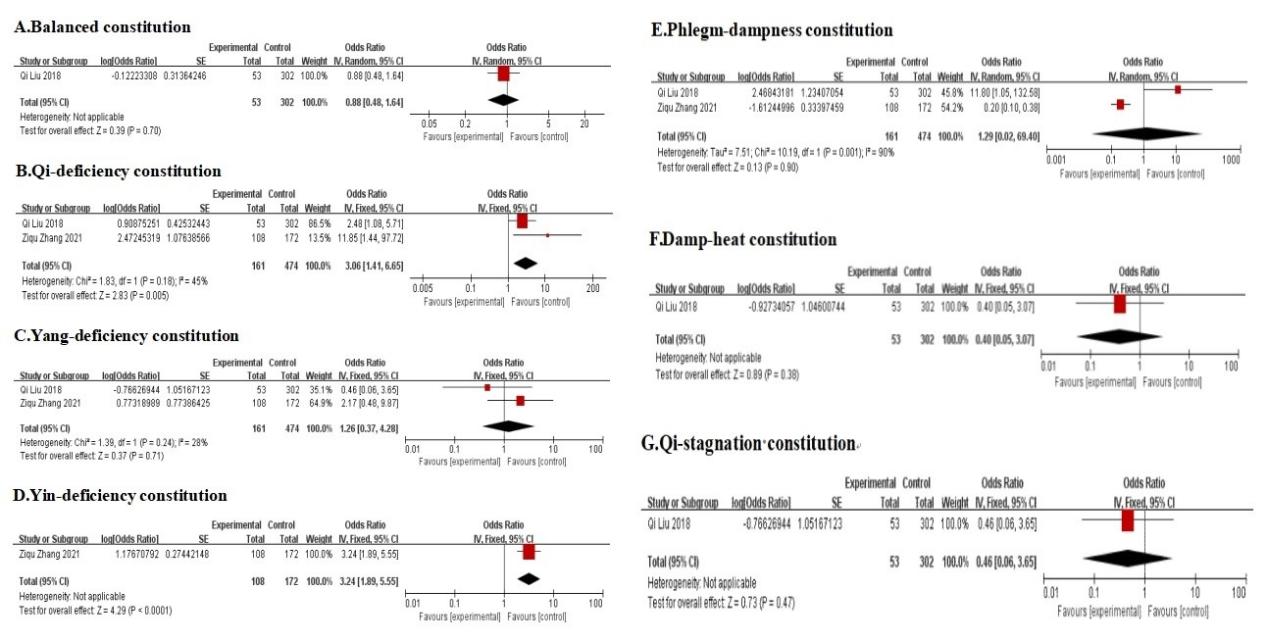
Supplementary Figure 17. **The forest plots** comparing TCM constitution distributions between hypotensive population and normal BP population.**

**
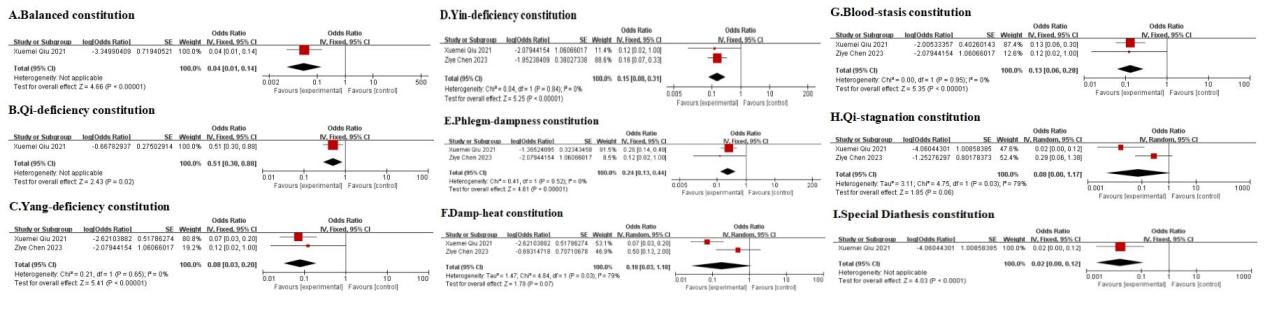
Supplementary Figure 18. **The forest plots of nine TCM** constitution distributions in ischemic cerebrovascular disease with hypoperfusion.**

**
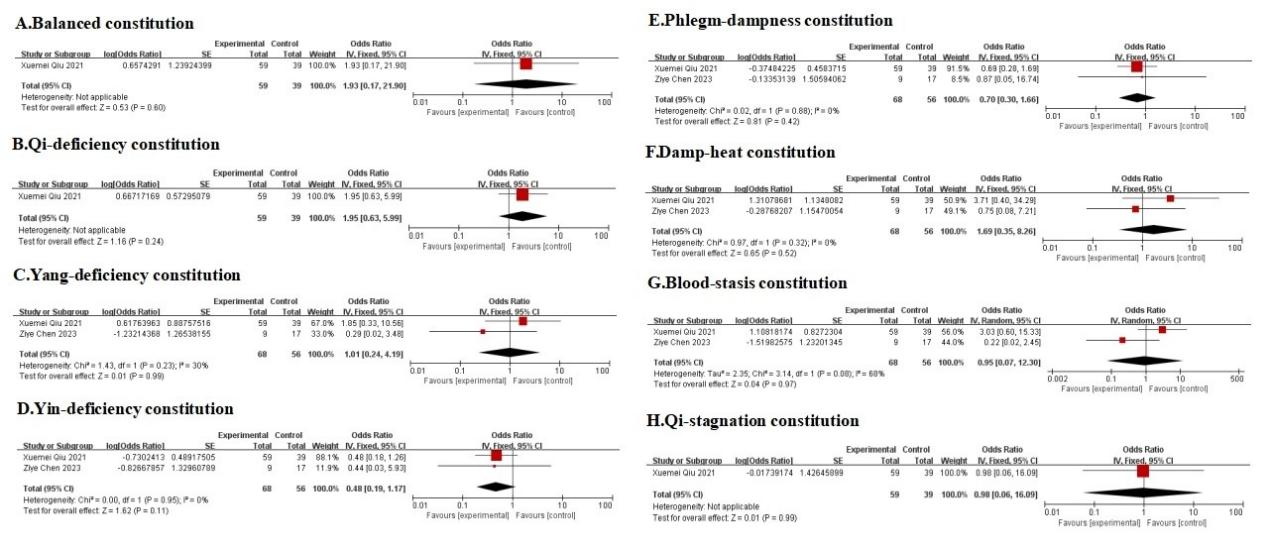
Supplementary Figure 19. **The forest plots** comparing TCM constitution distributions between ischemic cerebrovascular disease with hypoperfusion and ischemic cerebrovascular disease with normoperfusion.**
